# Comparative Pan-Genome Analysis of *Piscirickettsia salmonis* Reveals Genomic Divergences within Genogroups

**DOI:** 10.3389/fcimb.2017.00459

**Published:** 2017-10-31

**Authors:** Guillermo Nourdin-Galindo, Patricio Sánchez, Cristian F. Molina, Daniela A. Espinoza-Rojas, Cristian Oliver, Pamela Ruiz, Luis Vargas-Chacoff, Juan G. Cárcamo, Jaime E. Figueroa, Marcos Mancilla, Vinicius Maracaja-Coutinho, Alejandro J. Yañez

**Affiliations:** ^1^Facultad de Ciencias, Instituto de Bioquímica y Microbiología, Universidad Austral de Chile, Valdivia, Chile; ^2^Laboratory of Integrative Bioinformatics, Facultad de Ciencias, Centro de Genómica y Bioinformática, Universidad Mayor, Santiago, Chile; ^3^Centro FONDAP, Interdisciplinary Center for Aquaculture Research, Concepción, Chile; ^4^AUSTRAL-omics, Universidad Austral de Chile, Valdivia, Chile; ^5^Laboratorio de Patología de Organismos Acuáticos y Biotecnología Acuícola, Facultad de Ciencias Biológicas, Universidad Andrés Bello, Viña del Mar, Chile; ^6^Facultad de Ciencias, Instituto de Ciencias Marinas y Limnológicas, Universidad Austral de Chile, Valdivia, Chile; ^7^Laboratorio de Diagnóstico y Biotecnología, ADL Diagnostic Chile SpA., Puerto Montt, Chile; ^8^Laboratory of Integrative Bioinformatics, Instituto Vandique, João Pessoa, Brazil; ^9^Beagle Bioinformatics, Santiago, Chile

**Keywords:** gammaproteobacteria, piscirickettsiosis, salmonid rickettsial septicemia, comparative genomics, pan-genome, virulence factors, fish pathogen

## Abstract

*Piscirickettsia salmonis* is the etiological agent of salmonid rickettsial septicemia, a disease that seriously affects the salmonid industry. Despite efforts to genomically characterize *P. salmonis*, functional information on the life cycle, pathogenesis mechanisms, diagnosis, treatment, and control of this fish pathogen remain lacking. To address this knowledge gap, the present study conducted an *in silico* pan-genome analysis of 19 *P. salmonis* strains from distinct geographic locations and genogroups. Results revealed an expected open pan-genome of 3,463 genes and a core-genome of 1,732 genes. Two marked genogroups were identified, as confirmed by phylogenetic and phylogenomic relationships to the LF-89 and EM-90 reference strains, as well as by assessments of genomic structures. Different structural configurations were found for the six identified copies of the ribosomal operon in the *P. salmonis* genome, indicating translocation throughout the genetic material. Chromosomal divergences in genomic localization and quantity of genetic cassettes were also found for the *Dot/Icm* type IVB secretion system. To determine divergences between core-genomes, additional pan-genome descriptions were compiled for the so-termed LF and EM genogroups. Open pan-genomes composed of 2,924 and 2,778 genes and core-genomes composed of 2,170 and 2,228 genes were respectively found for the LF and EM genogroups. The core-genomes were functionally annotated using the Gene Ontology, KEGG, and Virulence Factor databases, revealing the presence of several shared groups of genes related to basic function of intracellular survival and bacterial pathogenesis. Additionally, the specific pan-genomes for the LF and EM genogroups were defined, resulting in the identification of 148 and 273 exclusive proteins, respectively. Notably, specific virulence factors linked to adherence, colonization, invasion factors, and endotoxins were established. The obtained data suggest that these genes could be directly associated with inter-genogroup differences in pathogenesis and host-pathogen interactions, information that could be useful in designing novel strategies for diagnosing and controlling *P. salmonis* infection.

## Introduction

*Piscirickettsia salmonis*, a Gram-negative and facultative intracellular bacterium, is responsible for salmonid rickettsial septicemia, or piscirickettsiosis (Fryer et al., 1990; Yañez et al., 2012). This disease causes high mortality rates in the three most important farmed salmonid species for the Chilean aquaculture industry, resulting in significant economic losses (Almendras and Fuentealba, 1997). *P. salmonis* was originally isolated and identified from diseased *Oncorhynchus kisutch* (Coho salmon) in southern Chile in 1989 (Bravo and Campos, 1989; Fryer et al., 1990), but this pathogen is not restricted to Chile. Indeed, piscirickettsiosis has subsequently been reported in Canada (Brocklebank et al., 1993), Ireland (Rodger and Drinan, 1993), Scotland (Grant et al., 1996), Norway (Olsen et al., 1997), southern USA (Olsen et al., 1997; Arkush et al., 2005), and Turkey (Öztürk and Altinok, 2014). *P. salmonis* causes systemic, chronic septicemia in fish, which exhibit a variety of internal symptoms, such as a discolored kidney, enlarged spleen, and pale liver with numerous nodules measuring 5–6 mm in diameter (Branson and Nieto Diaz-Munoz, 1991; Cvitanich et al., 1991). Despite research in several *in vitro* and *in vivo* models (Rozas and Enríquez, 2014), the mechanisms involved in *P. salmonis* pathogenicity are not entirely known. As such, current vaccination strategies, antibiotic treatments, and other biotechnological tools are not effective in controlling piscirickettsiosis (SERNAPESCA, 2016b). Furthermore, the continued high incidence of piscirickettsiosis outbreaks reported in Chile (SERNAPESCA, 2016a) is likely due to antibiotic resistance acquired by the pathogen (Cartes et al., 2016; Henríquez et al., 2016; Sandoval et al., 2016).

*P. salmonis* was originally grouped in the Rickettsiaceae family and reported as a rickettsia-like organism (Fryer et al., 1990, 1992). Further analyses of the nucleotide sequence and secondary structure of the 16S ribosomal RNA (rRNA) resulted in the relocation of *P. salmonis* into the Gammaproteobacteria in cursive class and Piscirickettsiaceae in cursive family (Fryer et al., 1992; Fryer and Hedrick, 2003). More recent research by Reid et al. (2004) using specific primers for 16S ribosomal DNA and the intergenic transcribed spacer (ITS) region of Irish and Scottish isolates revealed the potential existence of two geographically and phylogenetically separate *P. salmonis* groups. Similar approaches evidenced the potential existence of two representative genogroups isolated in Chile (Mauel et al., 1999; Bohle et al., 2014; Mandakovic et al., 2016; Otterlei et al., 2016), as specifically associated with the EM-90 and LF-89 strains. Differences within these genogroups have been reported in relation to geographic distribution, antibiotic susceptibility, and host specificity (Saavedra et al., 2017) Furthermore, these genogroups have been computationally compared using a limited number of fully sequenced genomes. For example, Bohle et al. (2014) used two complete genomes (strains PM15972A1, EM-90-like; and PM32597B1, LF-89-like) to genomically characterize the potential existence of genogroups. Additionally, Bravo and Martinez (2016) used 11 genomes (five complete and six draft genomes) to obtain a first approximation of the *P. salmonis* pan-genome. Nevertheless, these studies are limited in that they were performed with a restricted quantity of fully sequenced genomes. For instance, only one representative of the EM-90 strain was used by Bohle et al. (2014) and Bravo and Martinez (2016).

As with phylogenetic relationships and functional annotations, studies evaluating genomic structural divergences in virulence factors and metabolism within genogroups are scarce. Comparative genomic analyses of fully sequenced genomes are fundamental for defining the entire core- and pan-genomes of different isolates from the same species. The core-genome is defined as the entire repertoire of translated genes conserved among all isolates, with conservation suggesting that these genes are essential for basic bacterial survival. In turn, the pan-genome is the sum of the core genes and those within the “accessory genome,” i.e., the set of unique genes shared by or exclusive to certain strains. The presence of a pan-genome is associated with increased intra-species diversity, environment/host adaptations, and differentiations in pathogenic mechanisms (Tettelin et al., 2008). Several pan-genomic studies exist for different fish microorganisms, including marine pathogens such as *Aeromonas salmonicida* (Vincent and Charette, 2017), the *Alteromonas* ssp genus (López-Pérez and Rodriguez-Valera, 2016), and *Vibrio harveyi* (Espinoza-Valles et al., 2015). To define the entire core- and pan-genomes of an organism, a large quantity of fully sequenced genomes is essential. Advances in recent years mean that enough *P. salmonis* genome sequences are now available for pan-genome analysis.

The aim of this study was to differentiate and classify the number of *P. salmonis* genogroups by using phylogenetic and phylogenomic analyses, as well as by identifying structural and functional genomic divergences within the 19 fully sequenced genomes available for this pathogen. The obtained phylogenies and genomic structural divergences among the assessed *P. salmonis* strains strongly support a clear existence of two genogroups, termed herein as LF and EM. Moreover, pan-genome characterization identified genes involved in the *P. salmonis* core-genome that play roles in the life cycle, invasion, and pathogenic processes. Differences in virulence factors associated with established genogroups were elucidated by identifying and categorizing genogroup-exclusive genes. The detection of different genes potentially associated with pathogenicity, infection mechanisms, and host-pathogen interactions in the LF and EM genogroups is an important step toward understand the nature of variants of *P. salmonis* that needs to be explored in more detail in *in vivo* challenge studies.

## Materials and methods

### Genomes, gene predictions, and functional annotations

All publicly available *P. salmonis* genome sequences (25 in total: 19 full genomes, 6 draft genomes) were obtained from the National Center for Biotechnology Information (NCBI Resource Coordinators, 2016). Protein-coding genes were predicted using Glimmer v3.02 (Delcher et al., 1999). The predicted genes were annotated against the NCBI non-redundant (NCBI Resource Coordinators, 2016), Gene Ontology (GO) (Gene Ontology Consortium, 2015), and Swiss-Prot (UniProt Consortium, 2015) databases using DIAMOND (Buchfink et al., 2014). Metabolic pathways were recovered from the KEGG database (Kanehisa et al., 2016) using Blast2GO (Conesa et al., 2005). A summary of characteristics and the accession numbers for all publically available genomes are given in Table 1.

**Table 1:** General properties of all 19 *Piscirickettsia salmonis* with complete genome sequences available.

**Strain**	**# of contigs**	**# of proteins (This work)**	**# of proteins (NCBI)**	**Length (Mb)**	**GC content (%)**	**Accession number**	**Host**	**References**
AY3800B	1	2,496	2,909	3.19	39.7	CP013816.1	*S. salar*	–
AY3864B	1	2,493	2,908	3.19	39.7	CP013811.1	*S. salar*	–
AY6297B	1	2,498	2,908	3.19	39.7	CP013791.1	*S. salar*	–
AY6492A	1	2,489	2,889	3.04	39.7	CP013757.1	*S. salar*	–
AY6532B	1	2,538	2,895	3.19	39.7	CP013796.1	*S. salar*	–
LF-89 (ATCC VR-1361)	1	2,518	2,863	3.18	39.7	CP011849.2	*O. kisutch*	Pulgar et al., 2015
PM15972A1	1	2,479	2,909	3.06	39.8	CP012413.1	*S. salar*	Bohle et al., 2014
PM21567A	1	2,497	2,885	3.04	39.7	CP013762.1	*S. salar*	–
PM22180B	1	2,486	2,894	3.19	39.7	CP013801.1	*O. mykiss*	–
PM23019A	1	2,576	2,874	3.05	39.7	CP013768.1	*S. salar*	–
PM25344B	1	2,717	2,738	3.19	39.7	CP013821.1	*O. mykiss*	–
PM31429B	1	2,501	2,913	3.19	39.7	CP013806.1	*O. mykiss*	–
PM32597B1	1	2,488	2,897	3.19	39.7	CP012508.1	*O. kisutch*	Bohle et al., 2014
PM37984A	1	2,488	2,911	3.05	39.7	CP013773.1	*S. salar*	–
PM49811B	1	2,532	2,882	3.19	39.7	CP013781.1	*S. salar*	–
PM51819A	1	2,578	3,004	3.14	39.7	CP013778.1	*S. salar*	–
PM58386B	1	2,504	2,910	3.19	39.7	CP013786.1	*S. salar*	–
PSCGR01	1	2,510	2,866	3.17	39.7	CP013944.1	*O. kisutch*	–
PSCGR02	1	2,533	2,910	3.21	39.7	CP013975.1	*S. salar*	–

*All information was obtained from the NCBI database (NCBI Resource Coordinators, 2016). The Proteins numbers for each sequence was obtained by open reading frame predictions using Glimmer v3.02 (Delcher et al., 1999)*.

Only fully sequenced strains (19 total) were used for subsequent assessments. To facilitate comparative genomic analyses, the genome sequences of seven isolates (strains PM21567A, PSCGR02, PM22180B, PM25344B, PM31429B, PM49811B, and PM58386B) were modified to obtain the same initiation origin. Similarly, the reverse complementary sequences of three isolates (strains PM23019A, PM37984A, and PM51819A) were modified to match that in the majority of the included sequences.

### Phylogenetic and phylogenomic analyses

The phylogenetic relationships of ubiquitous, single-copy genes were obtained using genes *dnaK, groEL, recA, gyrA, gyrB, rpoB*, and *ftsZ*, the sequences of which were recovered from each analyzed genome. The same genes from *Francisella noatunensis* (Accession number CP018051) were used as an outgroup. The phylogenetic tree was obtained through TreeFinder (Jobb et al., 2004) and drawn using FigTree (http://tree.bio.ed.ac.uk/software/figtree/), applying the maximum likelihood method and a bootstrap with 50,000 iterations.

Phylogenomic analysis was performed according to Comas et al. (2007) and using all amino acid sequences available for the defined core-genome (see section Core- and Pan-Genome Definitions). First, the core proteins from each genome were concatenated in a unique file and ordered according to genome organization. All concatenated proteins were subjected to multiple alignment using the local MAFFT software (Katoh and Standley, 2013). Regions that were divergent, misaligned, or with a large number of gaps were eliminated using the Gblocks software (Talavera and Castresana, 2007), with default parameters. The phylogenomic tree was obtained through TreeFinder (Jobb et al., 2004) and drawn using FigTree (http://tree.bio.ed.ac.uk/software/figtree/), applying the maximum likelihood method and a bootstrap with 5,000 iterations.

### Core- and pan-genome definitions

All predicted genes were translated into proteins using TransDecoder within the Trinity Suite (Haas et al., 2013). For this, a minimum of 50 amino acids per protein was considered. The resulting proteins were used to identify orthologous groups among the assessed *P. salmonis* genomes, specifically through the GET_HOMOLOGUES tool (Contreras-Moreira and Vinuesa, 2013). All proteins were clustered using a combination of three different algorithms, i.e., bidirectional best-hit (Wolf and Koonin, 2012), COGtriangles (Kristensen et al., 2010), and OrthoMCL (Li et al., 2003). Orthologous proteins were defined as follows: at least 50% sequence conservation over 50% of the protein length (Tettelin et al., 2005). A final binary matrix was constructed based on the presence/absence of each protein across all *P. salmonis* strains. Finally, the core-genome was defined as the set of proteins shared by all strains, while the pan-genome was defined as the sum of the core-genome and the set of auxiliary (i.e., available in more than 1 and less than 19 genomes) and exclusive (i.e., available in only one genome) proteins.

The core- and pan-genomes, as well as estimated respective sizes and trajectories, were obtained using the method proposed by Knight et al. (2016) and the models/regression algorithms given by Tettelin and colleagues (Tettelin et al., 2005, 2008; Rasko et al., 2008). Curve fitting of the pan-genome was performed using a power-law regression based on Heaps' law [y = A_pan_x^Bpan^+C_pan_], as previously described (Tettelin et al., 2005, 2008; Rasko et al., 2008). Fitting was conducted with the PanGP software (Zhao et al., 2014), where *y* was the pan-genome size, *x* the genome number (i.e., 19), and *A*_*pan*_, *B*_*pan*_, and *C*_*pan*_ the fitting parameters. *B*_*pan*_ was equivalent to the γ parameter used by Tettelin et al. (2005, 2008) in estimating an open or closed pan-genome (Rasko et al., 2008). When 0 < *B*_*pan*_ < 1, the size of the pan-genome increases unboundedly with sequential additions of new genomes, thus indicating an open pan-genome. Conversely, when *B*_*pan*_ < 0 or *B*_*pan*_> 1, the trajectory approaches a plateau as further genomes are added, thus indicating a closed pan-genome. Curve fitting of the core-genome was performed using an exponential regression model [y = A_core_*e*^(Bcore x)^ + C_core_] (Tettelin et al., 2005, 2008; Rasko et al., 2008). New gene plots were derived from the pan-genome following the addition of each sequenced strain, thereby showing the number of novel “strain-specific” genes as a function of the number of strains (Knight et al., 2016).

Both the core- and pan-genomes were visualized through PanGP (Zhao et al., 2014), Qtiplot v0.9.9.11 (http://www.qtiplot.com/), and Anvi'o (Eren et al., 2015), using the above-mentioned binary matrix as an input.

### Virulence factors in pan-genome components

All protein sequences were aligned using BLASTp (Altschul et al., 1990) against the Virulence Factor Database for bacterial pathogens (Chen, 2004; Chen et al., 2016). The alignment parameters were an e-value of 1e-10, a minimum identity percentage of 40%, and a minimum coverage of 70% between the query and database sequences. Functional annotations were obtained from the categories and subcategories available in the “VFs.xls” file provided by the Virulence Factor Database.

### Ribosomal operons identification and genomic organization characterization

All 5S, 23S, and 16S rRNAs were predicted using the RNAmmer software (Lagesen et al., 2007). The genomic coordinates for each predicted rRNA were recovered and used for operon reconstruction and defining directionality in all *P. salmonis* strains. In a genomic context, for each operon was examined through manual explorations of all predicted coding genes available in the vicinity, specifically using a 1 kb window up- and downstream of the operon. The genomic coordinates and FASTA sequences were recovered for the first five adjacent protein-coding genes within the established window. Genomic comparisons among the different strains and genogroups were performed using BLASTn (Altschul et al., 1990).

### Growth kinetics

The bacterial isolation and growth kinetic was obtained using the method proposed by Saavedra et al. (2017) with isolates PM15972A1, PM21567A, PM23019A, PM32597B1, PM31429B, PM22180B, and type strain LF-89 [ATCC VR-1361]. PM15972A1 (EM-colony type) and PM32597B1 (LF-colony type) strains were used for visualization of the colony phenotypes using the ADL-PSA agar medium, corresponding to a solid derivative of the ADL-PSB medium (Henríquez et al., 2016) at 18°C for 6–8 days.

## Results

### Available genomic information for *Piscirickettsia salmonis*

The genome sequences of 25 different *P. salmonis* strains were found in the NCBI database (Table 1). Of these, 19 were fully sequenced strains and 6 were draft genomes. All strains presented similar GC-content (39.7–40%). The complete genomes ranged in size between 3.04 Mb (strain AY6492A) and 3.21 Mb (strain PSCGR02). The draft genomes ranged in length between 2.81 Mb (strain LF-89 ASSK00000000.2) and 3.53 Mb (AUSTRAL-005, Yañez et al., 2014). The number of predicted protein coding genes for the complete genomes ranged between 2,479 (strain PM15972A1) and 2,717 (strain PM25344B). These predictions differed from those available in NCBI records, which varied between 2,863 (strain LF-89 [ATCC VR-1361]) and 3,004 (strain PM51819A) predicted protein coding genes. This variation reflects the elimination of gene duplications in the present study, as achieved through clusterization (98% identity cutoff) with the CD-HIT software (Li and Godzik, 2006). Table 1 summarizes several key features for the 19 fully sequenced *P. salmonis* genomes.

### Phylogenetic analysis of 19 *Piscirickettsia salmonis* strains provides strong evidence for the existence of two different genogroups

**Figure 1:**
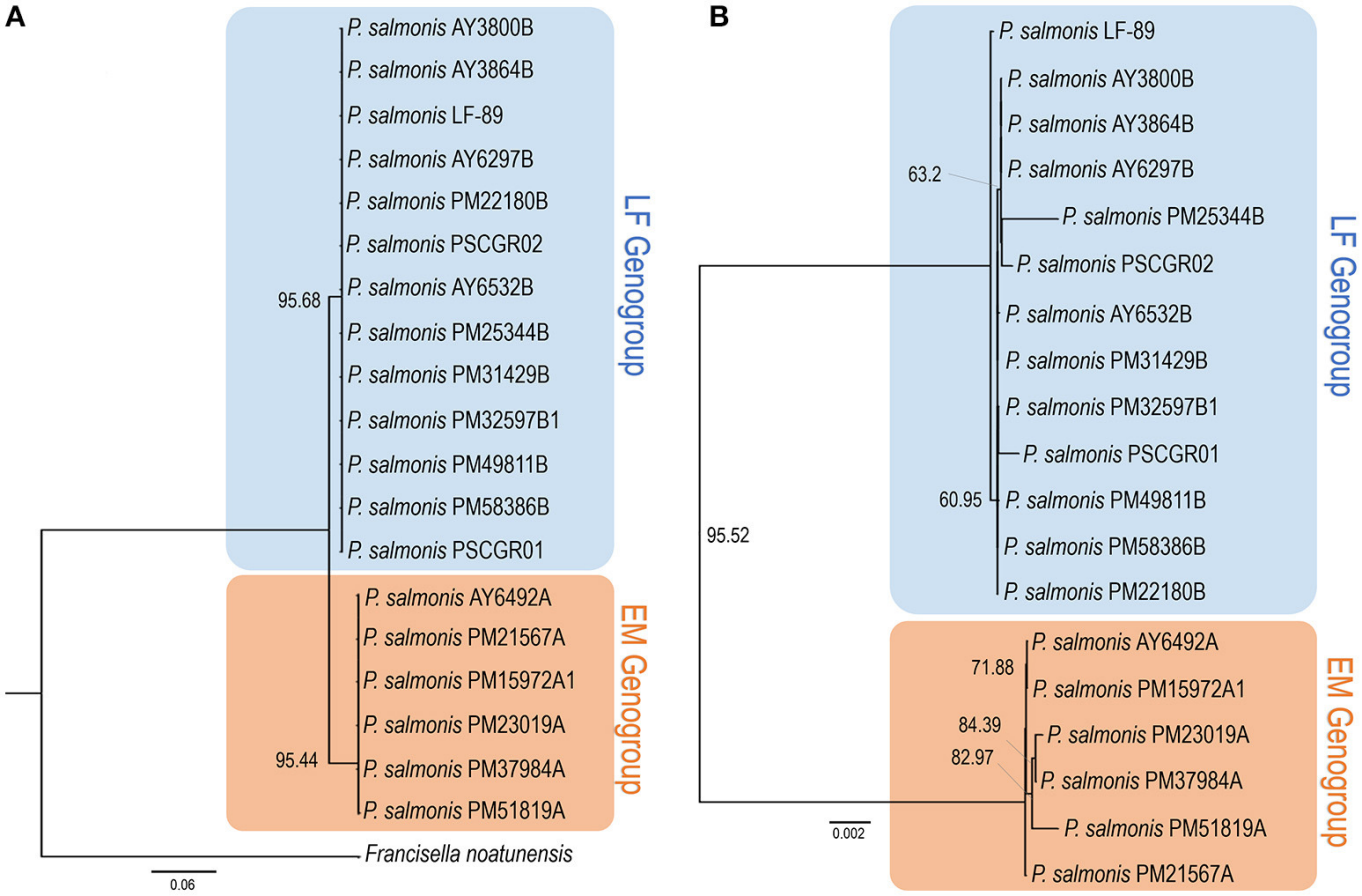
Phylogenetic and phylogenomics relationships. **(A)** Phylogenetic relationship of *Piscirickettsia salmonis* strains, according to the housekeeping genes shared by all complete genomes (i.e., *dnaK, groEL, recA, gyrA, gyrB, rpoB*, and *ftsZ*). **(B)** Phylogenomics tree generated based on a total of 1,732 protein sequences forming part of the *P. salmonis* core-genome. Trees were obtained through the TreeFinder software, using the maximum likelihood method and a bootstrap with 5,000 iterations. Color shading indicates to genogroup to which each stain belongs (blue: LF genogroup; red: EM genogroup). More information on strain characteristics is provided in Table 1.

These 19 fully sequenced strains were isolated from the Chilean salmon industry between 1989 and 2015. The percentages of strains isolated from different hosts were as follows: *Salmo salar*, 68.4%; *O. kisutch*, 15.8%; and *Oncorhynchus mykiss*, 15.8% (Supplementary Figure 1). To infer the phylogenetic relationship among all *P. salmonis* strains with an available complete genome sequence, a phylogenetic tree was constructed using seven ubiquitously conserved core genes (i.e., *dnaK, groEL, recA, gyrA, gyrB, rpoB*, and *ftsZ*) that were distributed in single copy along all genome sequences. The same genes from *F. noatunensis* were used as an outgroup. The resulting phylogenetic tree (Figure 1A) grouped the 19 *P. salmonis* isolates into two different groups, composed by 6 and 13 strains. Previous research has indicated the potential existence of two *P. salmonis* genogroups; these groups, representing strains derived from the EM-90 and LF-89 genogroups, have been previously termed A and B. Following this nomenclature, the name associated with each strain can be used to classify the strain into one of the two potential genogroups. The phylogenetic tree generated using seven single-copy genes showed a clear separation between both genogroups (Figure 1A), thus providing strong evidence for the existence of two genogroups within the 19 isolates with an available complete genome sequence. Draft genomes were not included in this tree as some constitutive genes were not found in the available contigs/scaffolds.

### Identification of core, auxiliary, and unique proteins within *Piscirickettsia salmonis* strains and genogroups

After the standardized prediction of protein coding genes within all isolates (Table 1), the sets of core, auxiliary, and unique proteins available in each genome were defined. The core-genome was defined as the set of proteins, i.e., translated from predicted coding genes, shared by all *P. salmonis* isolates. In turn, the accessory genome was defined as the set of proteins available in 1 or up to n-1 of the genomes (n: total genomes). A total repertoire of 3,463 proteins was identified in the *P. salmonis* pan-genome; of these, 1,732 proteins (50.01%) were part of the core-genome, and 1,731 proteins (49.99%) were part of the accessory genome. The set of core-genome proteins was used to perform a phylogenomic comparison of all 19 strains (Figure 1B). Phylogenomics analyses resulted in a tree with topology similar to obtained using conserved single-copy genes (Figure 1A), with two different subgroups once again clearly defined. These results strongly support the hypothesis that two genogroups exist for *P. salmonis*. The total number of estimated proteins obtained exclusively for isolates within the EM-90 genogroup (EM) was 273. Within the LF-89 genogroup (LF), 148 estimated proteins were found. It should be mentioned that these sequences belong to the core of each genogroups, so that these proteins are shared by all the strains of the genogroup to which they belong.

**Figure 2:**
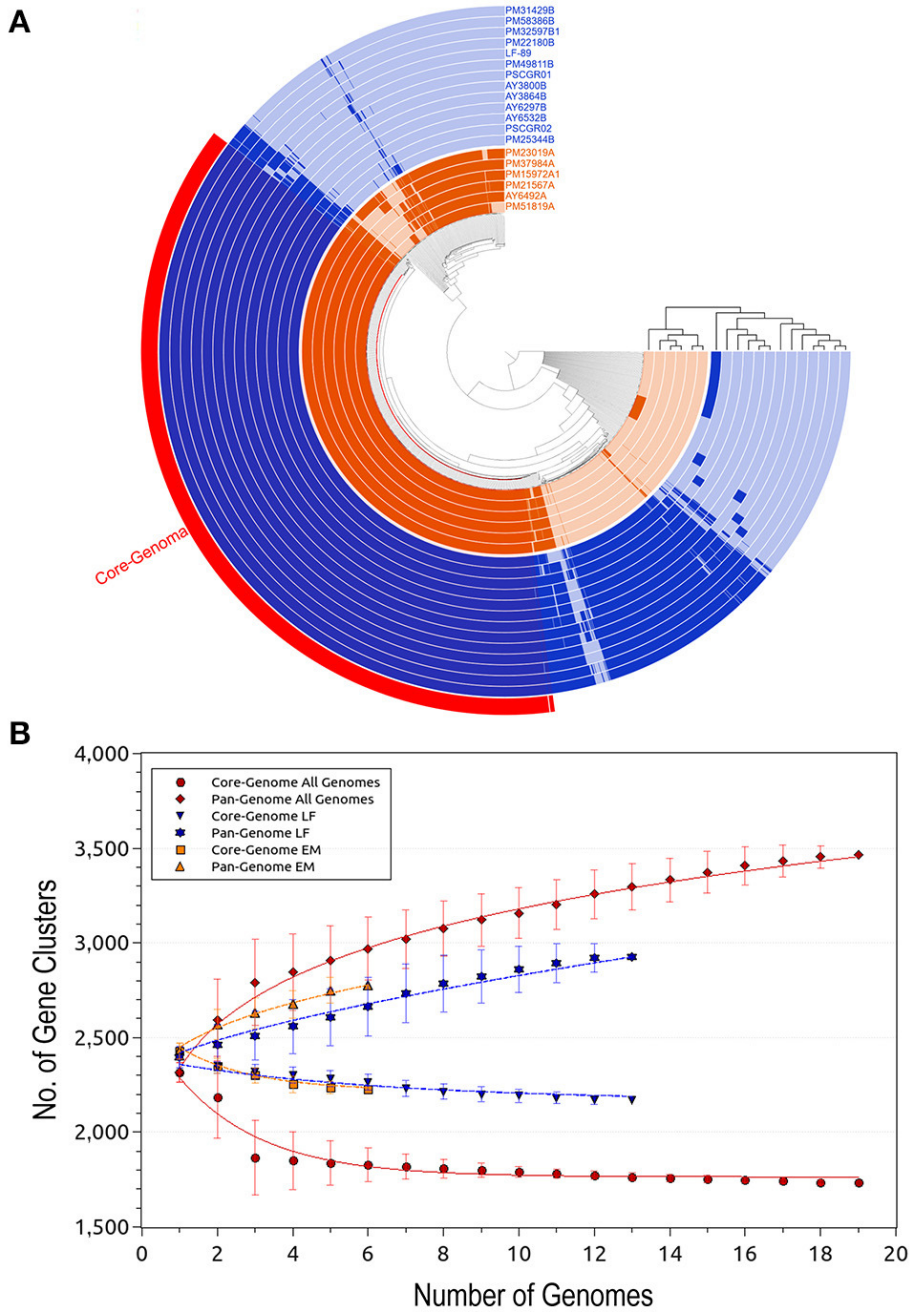
Predicted size of the *Piscirickettsia salmonis* pan-genome. **(A)** Comparative overview of *P. salmonis* pan- and core-genomes obtained through the Anvi'o tool (Eren et al., 2015). The *P. salmonis* core-genome is shown in red (1,732 proteins), while the different genogroups are indicated using blue (LF genogroup) and orange (EM genogroup). Each track indicates a genome, color variations (dark/light) indicate presence/absence of genes per genome. **(B)** Accumulation plots for the full *P. salmonis* pan- (red diamond) and core-genomes (red circle), as well as for the LF genogroup (blue inverted triangle [pan-genome] and star [core-genome]) and EM genogroup (orange squares [pan-genome] and triangles [core-genome]). Each plot point represents the mean value for any possible combination of gene clusters in the respective number of genomes (i.e. *P. salmonis* overall, 19 strains; LF genogroup, 13 strains; and EM genogroup, 6 strains). The curves represent a power law fitting of the data.

The Anvi'o (Eren et al., 2015) program was used to visualize the core- and pan-genomes (Figure 2A). A set of shared proteins was clearly shown across the 19 genomes. Similarly, the 1 and n-1 genomes presented a set of shared auxiliary and unique proteins. Anvi'o visualization also highlighted the protein sets available in each one of the *P. salmonis* genogroups. Cumulative curves, according to Heaps' law (Tettelin et al., 2008), were generated using PanGP (Zhao et al., 2014). Curves were generated for all 19 genomes, as well as separately for each genogroup (Figure 2B).

**Figure 3:**
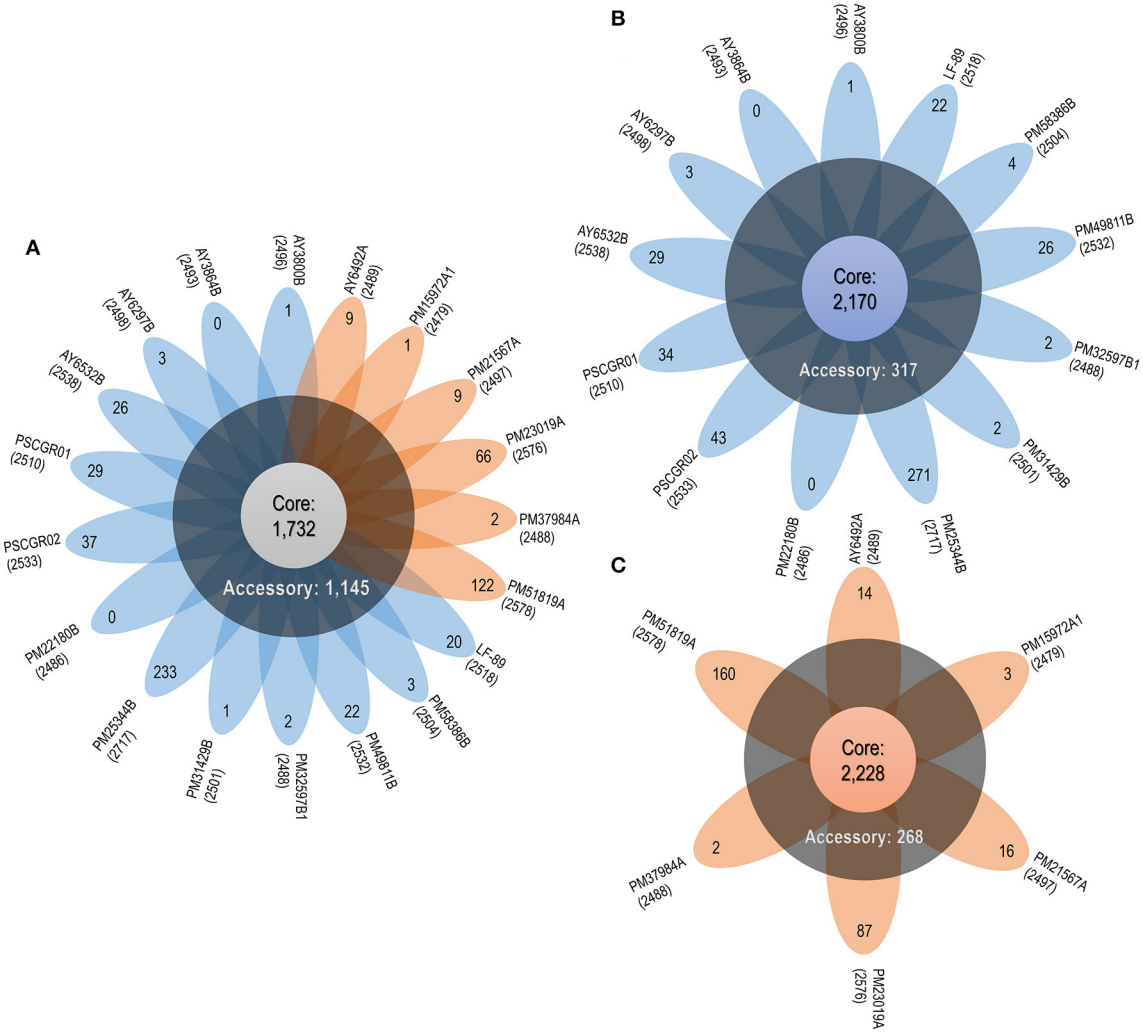
*P. salmonis* pan-genome. **(A)** Flower plot of all 19 *P. salmonis* strains showing the core-genome size (flower center), number of accessory genomes (around the flower center), and unique genes for each strain (flower petals). **(B)** Flower plot of the LF genogroup, showing the same information as in **(A)**. **(C)** Flower plot of the EM genogroup, showing the same information as in **(A)**. Numbers placed in parentheses below the name of each strain indicate the total proteins.

Apart for the set of 1,732 (50.01%) core proteins shared among all 19 isolates, comparative genomics analysis revealed a total of 1,145 (33.06%) accessory proteins and 586 (16.93%) unique proteins spread exclusively along independent strains (Figure 3A). One exception was the AY3864B strain, which did not present unique proteins. Interesting outliers were the PM25344B and PM51819A strains, which both presented a large number of unique proteins (i.e., 233 and 122, respectively). The number of unique proteins found in the remaining isolates ranged between 1 and 66 sequences.

Based on the obtained results, the *P. salmonis* pan-genome could be considered “open,” as supported by the γ parameter from Heaps' law (γ = 0.11). Furthermore, the *P. salmonis* pan-genome appears to be moderately expanding with the inclusion of new genomes (Tettelin et al., 2008). When separately investigating the core-genome for each of the *P. salmonis* genogroups, a total of 2,170 (74.21%) and 2,228 (80.20%) core proteins were found for the LF and EM genogroups, respectively (Figures 3B,C). In turn, the number of estimated auxiliary and unique proteins were respectively 317 (10.84%) and 437 (14.95%) for the LF genogroup and 268 (9.65%) and 282 (10.15%) for the EM genogroup. Worth noting, genes belonging to the core-genome generally maintained a similar genomic localization among all isolates. Indeed, the respective pan-genomes of the LF and EM genogroups could be classified as an “open” state, specifically when considering the γ parameter from Heaps' law (LF: γ = 0.66; EM: γ = 0.36).

### Functional similarities and divergences among *Piscirickettsia salmonis* strains and genogroups according to metabolic pathways and gene ontology terms

**Figure 4:**
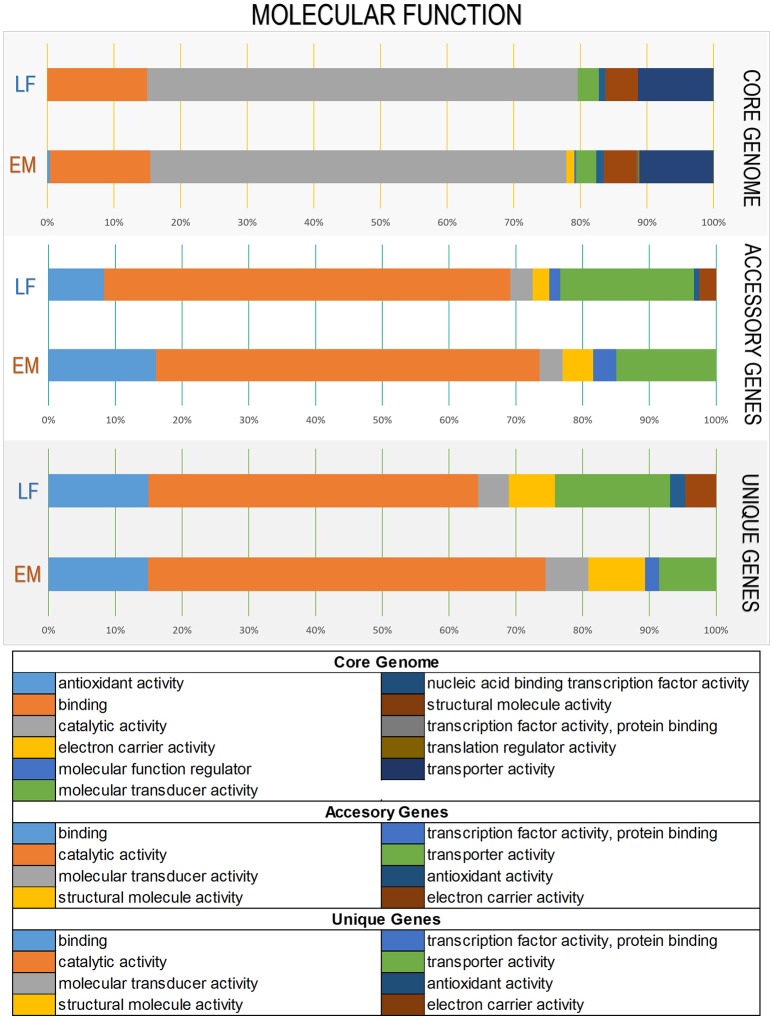
Variations in Gene Ontology functional annotations of the *P. salmonis* pan-genome. Shown are variations between the two genogroups in the “Molecular Function” Gene Ontology component. Variations were visualized using the annotation file (see Material and Methods) and the combined graph tool in Blast2GO (Conesa et al., 2005). The input data set were separated into the core-genome, accessory genome, and unique genes. Each bar represents a genogroup, and colors inside each bar represent a function of the genes contained in each section. Variations in the remaining Gene Ontology components are presented in Supplementary Figures 2, 3.

**Figure 5:**
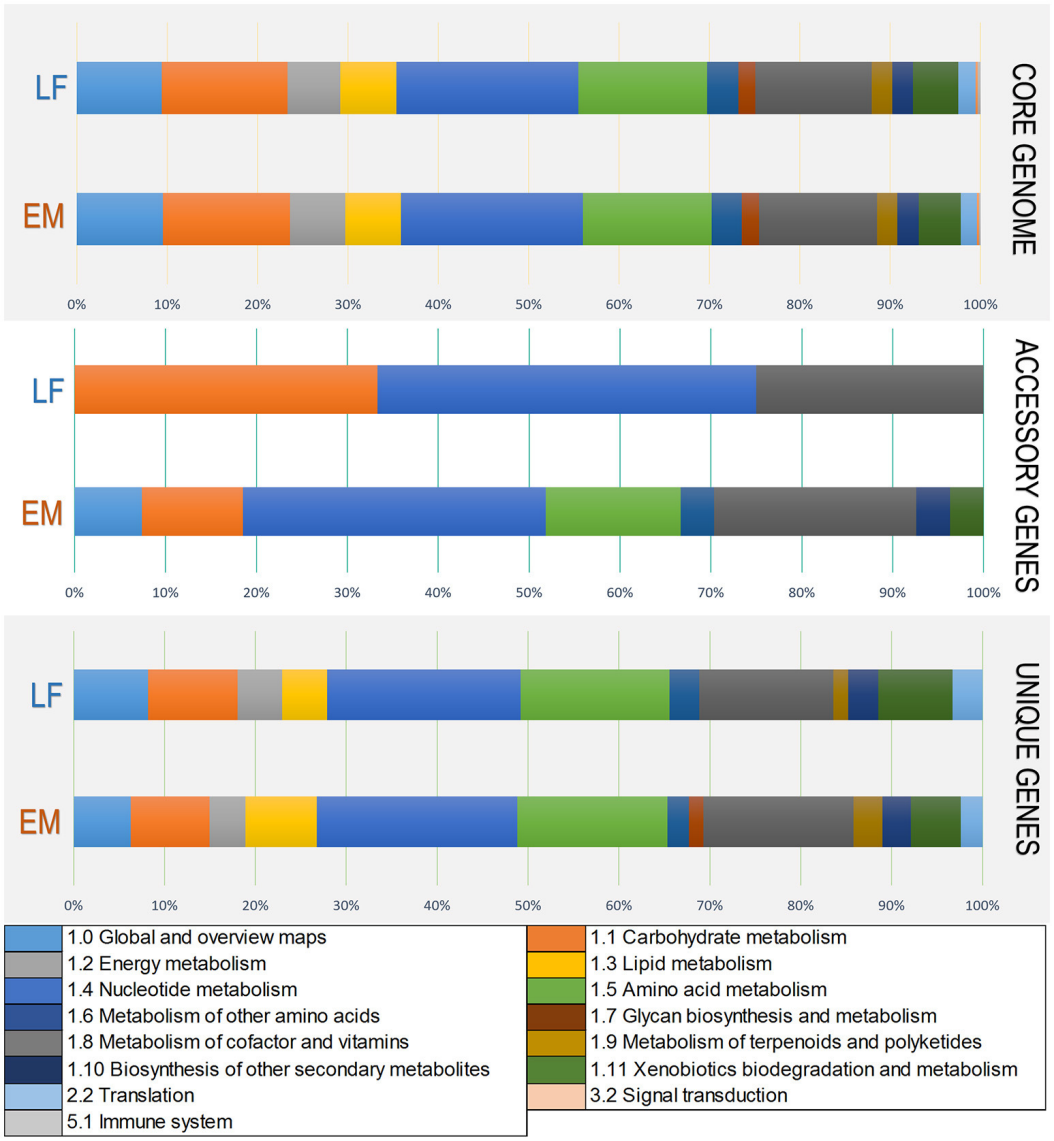
Variations in KEGG functional annotations of the *P. salmonis* pan-genome. Shown are variations between the two genogroups in KEGG categories. Variations were visualized using the annotation file (see Materials and Methods) and the KEGG annotation tool in Blast2GO (Conesa et al., 2005). The input data set were separated into the core-genome, accessory genome, and unique genes. Each bar represents a genogroup, and colors inside each bar represent a KEGG category for the genes contained in each section.

To elucidate functional variations in the core, accessory, and unique genes between the LF and EM genogroups, the functional categories of GO and KEGG annotations were compared. For gene ontology analysis, GO terms were successfully assigned to 1,450 out of 2,778 (52%) gene products in the EM genogroup; and 1,525 out of 2,924 (52%) gene products in the LF genogroup. KEGG annotation was applied to 1,325 out of 2,778 (49%) gene products in the EM genogroup and in 1,393 out of 2,924 (48%) gene products in the LF-89 genogroup (Table 2). GO analysis revealed differences between genogroups regarding the functional composition of core-genomes. The molecular function category (i.e., genes related to antioxidant activity, transcription factors, electron carrier activity, and translation regulation) were present in the EM genogroup but absent in the LF genogroup (Figure 4). Few differences were found for the cellular component category (Supplementary Figure 2); however, the biological process category showed minor variations in the biological-adhesion, growth, and rhythmic-process GO terms (Supplementary Figure 3). Regarding KEGG pathway analysis, the core-genomes of both genogroups were quite similar, showing a higher proportion of genes related to metabolic pathways for amino acids, nucleotides, lipids, carbohydrates, cofactors, and vitamins. Notably, the accessory genome for the EM genogroup presented several genes from categories related to amino acid metabolism, xenobiotic degradation, and the biosynthesis of secondary metabolites, but these genes were absent in the LF genogroup (Figure 5).

**Table 2:** Total annotations of each pan-genome component against the Gene Ontology (Gene Ontology Consortium, 2015) and KEGG (Kanehisa et al., 2016) Databases.

		**Total sequences**	**Annotated with GO**	**Annotated with KEGG**	**KEGG pathways**
LF genogroup	Core-genome	2,170	1,277	1,229	111
	Accessory genes	317	107	27	15
	Unique genes	437	141	127	52
EM genogroup	Core-genome	2,228	1,300	1,269	111
	Accessory genes	268	58	12	6
	Unique genes	282	92	61	36

*All information used in the annotation was obtained from previous analyses. Shown are the total sequences in each component of the pan-genome (i.e., core-genome, accessory genes, and unique genes), total sequences annotated against the Gene Ontology database, total sequences annotated against the KEGG database, and the number of KEGG pathways involved in each annotated sequence*.

**Figure 6:**
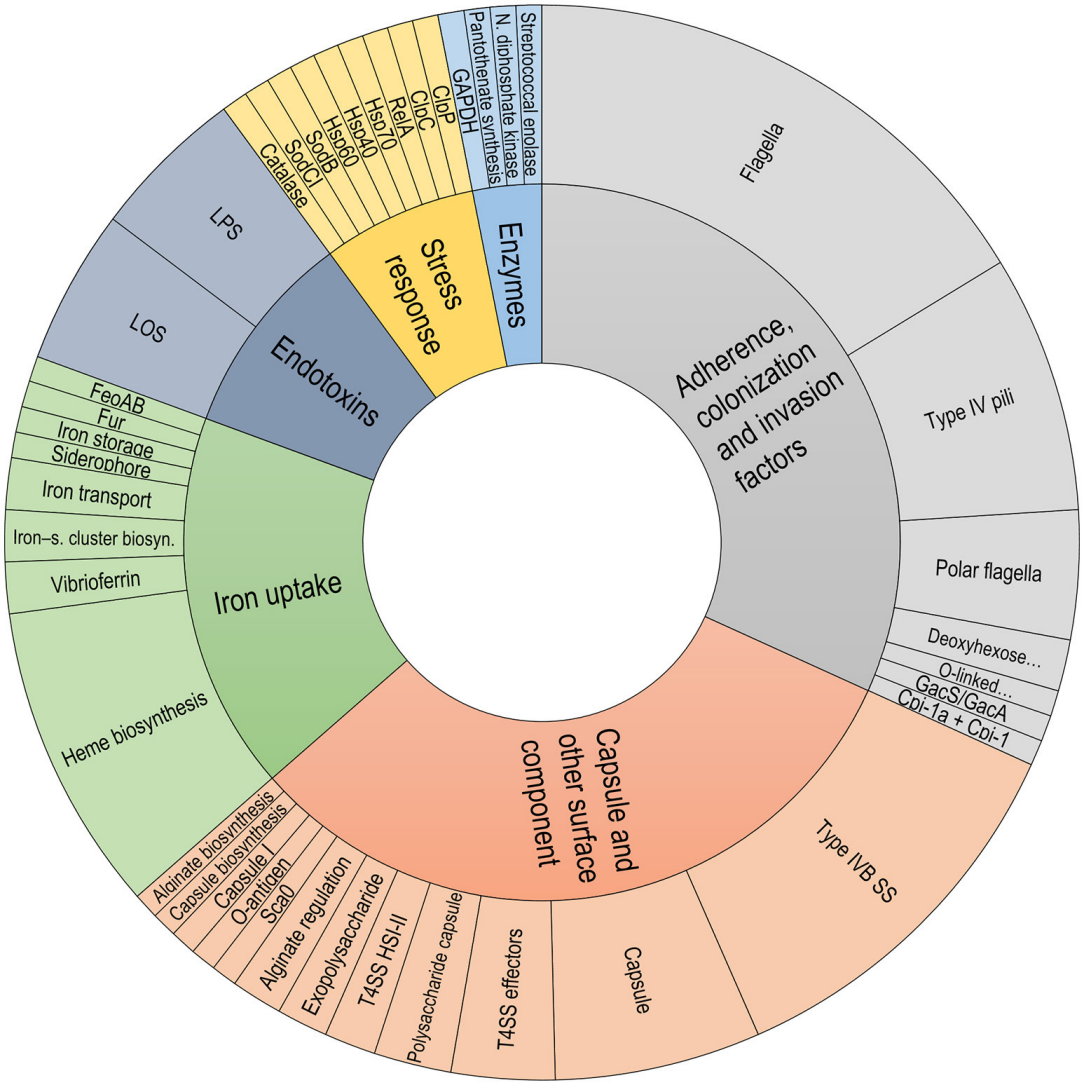
Classification of virulence factors in *the P. salmonis* core-genome. Classification was performed using data extracted from the Virulence Factor Database description file.

Common components related to virulence factors were found among the *P. salmonis* strains. In the *P. salmonis* core-genome, important gene groups were identified with some degree of shared pathogenicity. These genes were grouped into different categories and subcategories within the Virulence Factor Database, such as “Adherence, colonization and invasion factors,” “Capsule and other surface components,” “Endotoxins,” “Iron uptake,” “Stress response,” and “Enzymes” (Figure 6). Interestingly, unique virulence factors were identified for each genogroup, with these factors forming part of the respective core-genomes. These unique virulence factors included the Patatin-like protein, transposases, and glycosyltransferase group 1 in LF genogroup. Unique virulence factors in the EM genogroup included Listeria Adhesion Protein, fic/DOC protein and putative glycosyltransferase. Functional annotations with virulence factors from the Virulence Factor Database are listed in Supplementary Table 1.

### Comparative genomics reveals divergent regions, suggesting different organizations

**Figure 7:**
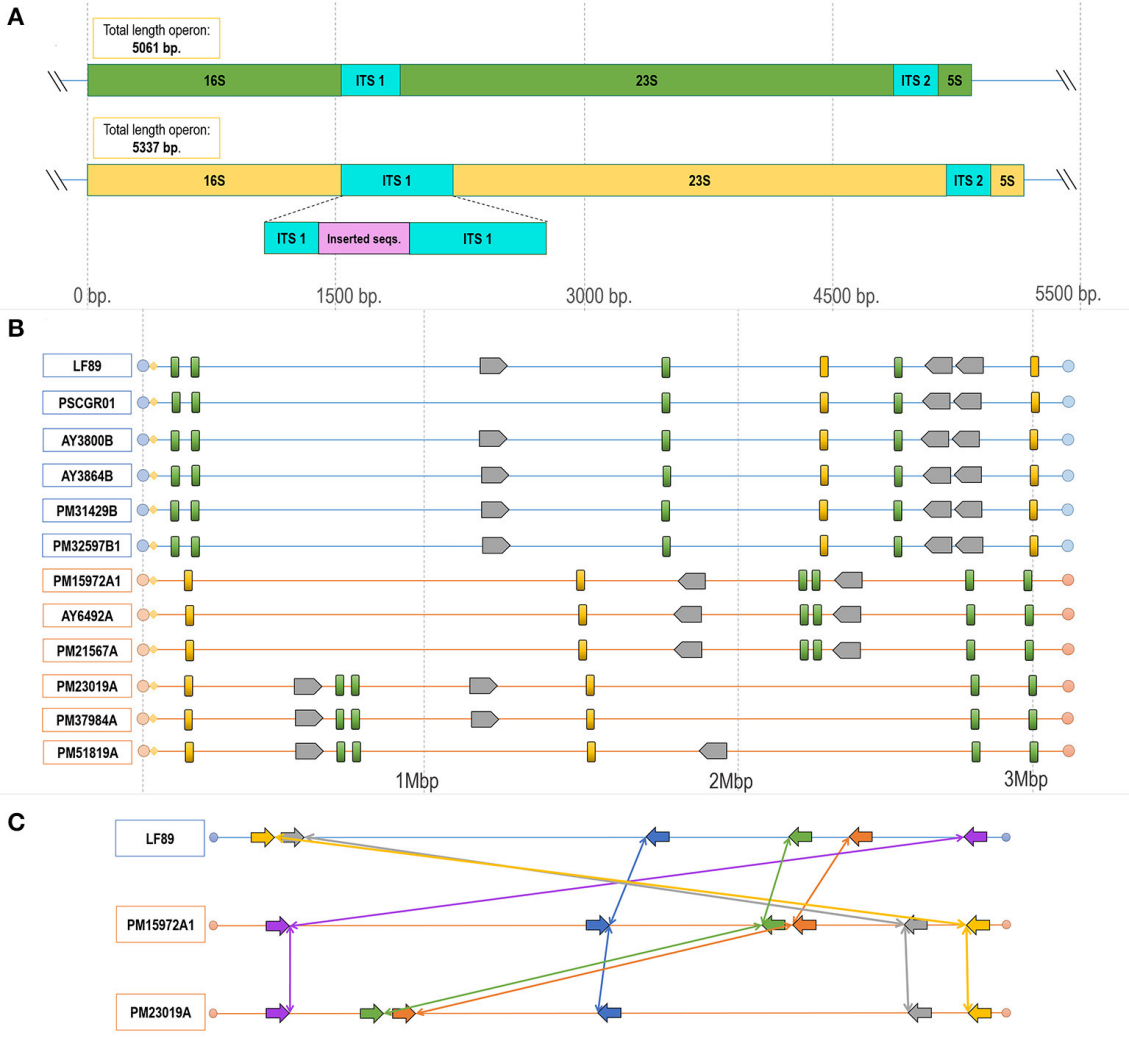
Configuration and rearrangement of operons in the different *P. salmonis* genogroups. **(A)** Operons A and B in the *P. salmonis* LF-89 strain (Accession Number CP011849.2). Differences in length existed between the operons, with operon A containing 5,061 nt and operon B 5,337 nt. This size difference was given by the insertion of a sequence fragment into the ITS1 region. **(B)** Structural genomic organization of ribosomal operons in all *P. salmonis* strains with an available complete genome sequence (LF genogroup strains: blue lines; EM genogroup strains; orange lines). Operon A (green blocks) varied in length between 5,061 and 5,064 bp. Operon B (yellow blocks) varied in length between 5,335 and 5,338 bp. The number of gene cassettes (gray blocks) of the Dot/Icm type IVB secretion system varied by genogroup. **(C)** Locations and directions of the operons, with reorganizations indicated by connecting colored lines and arrows. These sequences were ordered according to the distribution of the contiguous genes distributed near each operon, as detailed in Supplementary Figure 5, Supplementary Table 3.

Comparative genomics analysis revealed structural differences along the *P. salmonis* genome. Structural differences included (i) variable lengths of whole genome sequences; (ii) the presence/absence of gene sets (Table 1, Figures 4, 5); (iii) gene rearrangements at specific locations; and, for some isolates, (iv) foreign sequences inserted into highly conserved regions (Figure 7C). Additional exemplary divergent regions within strains and genogroups were presented by using the genomic organization of ribosomal operons and the gene cassette *Dot*/*Icm* type IVB secretion system.

Manual exploration of the genomic context for the ribosomal operons from all strains revealed divergences within the ITS regions, which were characterized by a prevalence of two types of ITS sequences between the 16S and 23S rRNA genes. These divergences were spread over all *P. salmonis* genomes. Furthermore, the number of copies and the genomic organization of these operons varied among strains and, especially, genogroups. Comparisons of both operon types resulted in the identification of potential SNPs (Supplementary Figure 4), and one operon was notable for presenting an insertion of 276 nucleotides. These two operon types were classified according to length and termed herein as operon A (5,061–5,064 bp) and operon B (5,335–5,338 bp), which contained the inserted sequence (Figure 7A). An alignment between both ITS sequence types is given in Supplementary Figure 4. Comparisons of genomic organization between operons A and B among all strains and genogroups revealed that, in all LF and EM isolates, operon A always presented four copies and operon B always presented two copies (Figure 7B). Different rearrangements of these operon sequences in the genomic organization were observed within both genogroups, but, especially, in isolates from the EM genogroup (Figures 7B,C). Chromosomal inversions and translocations were also identified inside the EM isolates, thus emphasizing potential sequence divergences (see Supplementary Table 2 for genomic coordinates of each ribosomal operon). Chromosomal rearrangements and organizations were obtained by exploring the distribution of five consecutive genes located within a 1 kb window on both sides of each operon (Supplementary Figure 5, Supplementary Table 3). *In vivo* observations revealed that LF genogroup isolates (i.e., LF-89, PM32597B1, PM31429B, and PM22180B) were able to grow at 18°C, but not at 22°C. By contrast, EM genogroup isolates (i.e., PM15972A1, PM21567A, and PM23019A) grew at both temperatures (Supplementary Figure 6). This differential phenotypic capacity to adapt and grow at different temperature was could be correlated with sticky and mucoid phenotypes of *P. salmonis* isolates in the agar colony (Supplementary Figure 7).

Genomic divergences among isolates and genogroups were also found in regions from the gene cassette *Dot/Icm* type IVB secretion system, which is composed by *icmK, icmE*, i*cmG, icmC, dotD, dotC, dotB, icmT, icmO, icmP, icmJ, icmW, icmB, dotA, icmL*, and *icmV*. This group of genes has a conservative organization in distribution order along the gene cassette. Three and two copies of this genomic cassette were respectively found in LF and EM genogroup isolates (Figure 7B). Additionally, the *icmT* gene was exclusively found in two LF genogroup genomes (i.e., isolates AY6532B and PSCGR02).

## Discussion

Different studies using 16S rRNA-based phylogenetic approaches suggest the existence of two clusters among *P. salmonis* strains, as associated with the representative LF-89 and EM-90 strains (Mauel et al., 1999; Bohle et al., 2014; Mandakovic et al., 2016; Otterlei et al., 2016). Differences between these genogroups include geographic distributions, antibiotic susceptibilities, and host specificities (Saavedra et al., 2017). Recently, Bravo and Martinez (2016) reported comparative genomics results supporting the existence of these two genogroups. More specifically, seven draft genomes and five complete genome sequences (LF genogroup: 4 and EM genogroup: 1) were used to describe the *P. salmonis* core-genome, with a total of 1,683 proteins. In the present study, comparative genomics analyses were performed using 19 complete genome sequences (LF genogroup: 13 and EM genogroup: 6). Phylogenetic analysis was conducted using seven conserved single-copy genes. In turn, whole core-genome phylogenomics analysis used the core proteins from all 19 genomes, resulting in strong evidence for the existence of two genogroups. This analysis of all 19 isolates also provided a comprehensive representation of the *P. salmonis* core- (1,732 proteins) and pan-genomes (3,463 proteins), as well as of the genogroup-specific core- (LF genogroup: 2,170 proteins and EM genogroup: 2,228 proteins) and pan-genomes (LF genogroup: 2,924 proteins and EM genogroup: 2,778 proteins).

The “open” or “close” state of pan-genome it will depend of the capacity of acquire exogenous DNA (Medini et al., 2005; Diene et al., 2013), This phenomenon is commonly seen in species living in bacterial communities, tend to have large genomes and an open pan-genome, a high horizontal gene transfer range and several ribosomal operons (Georgiades and Raoult, 2011; Diene et al., 2013). Exist evidence that the pathogen *P. salmonis* generates biofilm in stages of stress (Marshall et al., 2012; Albornoz et al., 2017), which are essential for the organization of bacterial communities (Filloux and Vallet, 2003). The obtained results indicate that the *P. salmonis* pan-genome has an open state. Any efforts to “close” the pan-genome should focus on obtaining new complete genomes from various isolates. The higher number of core proteins found in the present study might be due to the use of more complete genomes than have been analyzed in prior reports. Indeed, comparative genomic assays using incomplete genomes run the risks of only detecting partial gene sequences, which may erroneously be classified as novel gene families, or not detecting certain genes at all. For instance, publicly available genomic *P. salmonis* annotations present potentially erroneous protein-coding gene predictions. Several incomplete genes, i.e., containing only partial fragments of protein-coding genes, are spread across all available incomplete genome sequences (Denton et al., 2014). The presence of these small gene fragments could be the result of inherent genomic traits (e.g., chromosome rearrangements) (Darling et al., 2008; Jackson et al., 2011), gene duplications (Jackson et al., 2011), or bioinformatics-based biases in errors (e.g., assembling complications, false positive gene predictions) arising from the incompleteness of these genome sequences (Schnoes et al., 2009; Denton et al., 2014).

The annotation process of all predicted genes revealed functional similarity and divergences within strains and genogroups according to KEGG metabolic pathways and gene ontology categories. Previous studies confirm that metabolic pathways of nucleotides, followed by carbohydrates, cofactors and vitamins, amino acids and lipids are highly conserved in *gammaproteobacteria* (Poot-Hernandez et al., 2015). These same metabolic pathways are highly conserved in both *P. salmonis* genogroups, as can be seen in the “Core-genome” item in Figure 5.

On the other hand, a difference might exist at the colony-phenotype level for both LF-like and EM-like *P. salmonis* strains (Bohle et al., 2014). For instance, LF-89-like isolate colonies present a stick phenotype (Supplementary Figure 7A) when left for several days on culture plates, whereas EM-90-like strain colonies present a mucoid phenotype (Supplementary Figure 7B). This phenotype difference between EM and LF may be due to the varied distribution of genes encoding glycosyltransferase in association with lipopolysaccharides formation. More specifically, distinct protein sets appear to be preferably distributed within genogroups. Moreover, studies by Saavedra et al. (2017) demonstrate that this differential phenotype is confirmed by differential growing kinetics at 18 and 22°C. Interestingly, only the EM genogroups is able to grow at 22°C whereas the LF genogroups did not grow (Saavedra et al., 2017). In that work, they use different field isolated that belong to both genogroups. Similar analysis of the fully sequence genomes strains used in this *in silico* analysis shown the same results indicating that both genogroups have divergences that change the ability to grow at different temperature (Supplementary Figure 6).

This is the first phylogenomic and structural genomics study suggesting the existence of two *P. salmonis* genogroups (denominated LF and EM), as well as at least two subgroups within the EM genogroup (determined via phylogenomic and structural localization of the ribosomal operon in the chromosome). These genomic analyses are interesting in light of previous studies and particularly the recent work by Rozas-Serri et al. (2017), a study in which controlled challenge assays resulted in different clinical signs for the EM and LF strains. These differences demonstrate the need for genetically understanding the differences between the genogroups characterized in the present study. The data-supported existence of these genogroups represents material and basic knowledge that might impact on vaccine development where it will be important to show if there are immunogenic differences between EM and LF genogroups, particularly since current vaccines are mainly based on LF variants. Additionally, these findings should also encourage comparison of EM and LF genogroup with regard to pathogenic differences both *in vitro* and *in vivo*. Moreover, the study of multiple genomes of pathogen species combined with reverse vaccinology can let to identify common antigens that could potentially protect against more than one subgroup of *P. salmonis* and other bacterial species. Further studies need to be done in order to stablish the potential of this vaccine technology (Gourlay et al., 2017).

Common virulence factors were found in the *P. salmonis* core-genome, and the identified *P. salmonis* virulence factors were grouped into the following six functional categories: (i) endotoxins, including lipopolysaccharides and lipooligosaccharides; (ii) iron uptake, including Fur, siderophores, vibrioferrins, and Heme biosynthesis; (iii) capsule and other surface components, including capsule biosynthesis, exopolysaccharides, the type IVB secretion system, and the type VI secretion system; (iv) adherence, colonization, and invasion factors, including type IV pili, flagella, and polar flagella; (v) enzymes, including Pantothenate biosynthesis and GAPDH; and (vi) stress response category, including Hsp40, Hsp60, Hsp70, and ClpP. Interestingly, some virulence factors were genogroup-specific. For example, LF isolates evidenced an exclusive presence of the Patatin-like (*Pat*) protein, which exhibits phospholipase activity, which are associated with host entry, phagosomal escape, and host lysis for spreading cell-to-cell (Rahman et al., 2013); transposases, which are responsible for transposons movement (Jackson et al., 2011); and a glycosyltransferase group 1, which catalyzes the transfer of glucose residues involved in synthesizing the outer core region of lipopolysaccharides (Ünligil and Rini, 2000; Leipold et al., 2007). In turn, EM strains uniquely evidenced the Listeria Adhesion Protein, that interacts with the Hsp60 host-cell receptor to promote bacterial adhesion in *Listeria* ssp. (Jagadeesan et al., 2010); the fic/DOC family protein, a toxin that mediates post-translational modifications of host cell proteins, thus specifically interfering with signaling, cytoskeletal processes, or translation (Roy and Cherfils, 2015); and a putative glycosyltransferase, which is essential for O-polysaccharide synthesis (Dabral et al., 2015). These genogroup-specific virulence factors could grant special traits. In a similar context, specific *Rickettsia typhi* groups possessing genes encoding for the Patatin-like protein (Rahman et al., 2013) have advantages against the host during the infection process.

Gene duplication is an important factor for the formation and evolution of bacterial genomes (Romero and Palacios, 1997). Six complete rRNA operons were found in all presently analyzed *P. salmonis* genomes. All six rRNA operons shared the same basic structure (16S–ITS−23S−5S), and two carried a 276-nucleotide insert on the respective ITS regions. Differences in the genomic organization of rRNA operons were able to associate both types of ribosomal operons with the two defined *P. salmonis* genogroups (LF or EM). In a recently published study, Saavedra et al. (2017) described marked differences between genogroups, with the EM strains being able to grow in a wider temperature range and having lower nutritional demands. Another structural divergence within genogroups was in the organization of ribosomal operons. The rRNA operon is composed by the 16S, 23S, and 5S rRNA genes, as well as by an ITS between the 16S and 23S genes (Lecompte, 2002). The rRNA operon is essential for protein biosynthesis and, since the 1980s, has been the gold standard for bacterial identification and for establishing evolutionary relationships between bacterial species (Woese, 1987; Hashimoto et al., 2003; Pei et al., 2010). rRNA operon analyses have also helped elucidate how different bacteria grow and thrive. Indeed, the rRNA operon copy number is considered a measure of growth rate and/or adaptability, as organisms with a higher number of rRNA operons can achieve faster growth under certain conditions (Klappenbach et al., 2000; Yano et al., 2013). Despite this, not all rRNA operons are transcribed at the same rate, and in *Escherichia coli*, the loss of certain operons does not affect growth (Condon et al., 1995). In turn, diverse promoters of rRNA operons are differentially regulated under stringent conditions in *Bacillus subtilis* (Samarrai et al., 2011), while in *Vibrio cholerae*, rearrangements of constitutive genes, including ribosomal operons, increase the bacterial transcription rate if able to move to a location closer to the replication origin (Couturier and Rocha, 2006). Organisms with more chromosomal rRNA operons might have a higher potential for “fine-tuning” expressions to adapt to different environmental conditions (Condon et al., 1995). This would reinforce the postulation that while some operons are important for growth rate or physiology, others have more adaptive purposes. This mechanism also accounts for the quantity of type IVB secretion system clusters spread over the *P. salmonis* genomes, as well as for the degree of observed synteny. Bacterial genomes may carry multiple divergent copies of T4BSS, which is reflective of functional diversity (Gillespie et al., 2015). Intriguingly, this was not the case for *P. salmonis* T4BSSs, which was found to present copy-number divergences mainly within genogroups. For instance, *Dot/Icm* T4BSS in LF isolates was predominantly distributed in three copies, each composed by 16 genes. Exceptions were the PSCGR01 isolate, distributed into two copies. In EM strains, the *Dot/Icm* secretion system was distributed in two copies, each composed by 16 genes. The expression of *Dot/Icm* T4BSS genes has been detected in *P. salmonis*, supporting participation in transcriptional activity (Gómez et al., 2013). The data suggest that the chromosomal divergences in genomic localization and quantity of genetic cassettes found in the *P. salmonis* genome, indicated that is a common driving force for the differential evolution of LF and EM genogroups.

## Conclusions

Comparative genomic analysis of 19 fully sequenced *P. salmonis* genomes provided evidence for the existence of two genogroups, as linked to the EM and LF type strains. This hypothesis was further supported by phylogenetic and phylogenomics analysis; and chromosome distribution assessments for the ribosomal and type IVB secretion systems. Each genogroup presented differences in DNA rearrangements, with more divergences observed within isolates from the EM genogroup. Divergences included variations in ribosomal operons, suggesting a potential evolution into two novel subgroups. On the other hand, the *P. salmonis* pan-genome was determined in an open state, which might account for the observed genomic variability. Pan-genome analysis also allowed for identifying auxiliary and unique genes specific to each strain and genogroup. These genes might provide diverse advantages to the respective genogroups.

To the best of our knowledge, the present study is the most comprehensive comparative genomics characterization of complete genome sequences for *P. salmonis*. The characterized divergences and similarities represent a notable contribution toward understanding the biology and evolution of *P. salmonis*. Additionally, the obtained data serve as a starting point in developing innovative methodologies for discriminating between *P. salmonis* strains and genogroups, which will be useful for more specifically diagnosing infection and for selecting potential candidate genes that could be applied in the control of piscirickettsiosis.

## Author contributions

VM and AY supervised the study. GN, CM, VM, and AY participated in the design and discussion of the research. GN, CM, and DE performed bioinformatics analyses. GN, VM, AY, PR, LV, JC, JF, and MM contributed to the acquisition, analysis, and interpretation of data for the work. GN, PS, CO, VM, and AY drafted the manuscript. All authors read and approved the final manuscript.

### Conflict of interest statement

The authors declare that the research was conducted in the absence of any commercial or financial relationships that could be construed as a potential conflict of interest.

## Supplementary material

Supplementary Table 1 Virulence factors identified in the core-genome.

Supplementary Table 2 Information on ribosomal operons in the 19 complete genomes of *P. salmonis*.

Supplementary Table 3 Annotation of the genetic neighborhood of each ribosomal operon. Each ID corresponds a gene represented in the Supplementary Figure 5.
