# Supplementary material for: Comparative Pan-Genome Analysis of Piscirickettsia salmonis Reveals Genomic Divergences within Genogroups
Source: Front Cell Infect Microbiol. 2017 Oct 31;7:459. doi: 10.3389/fcimb.2017.00459 (PMC5671498; doi:10.3389/fcimb.2017.00459)
Supplement: Supplementary Table 1 — Virulence factors identified in the core-genome. [file Table1.DOCX]

**Supplementary Table 1**

| Virulence Factor category | Protein name | Description | Virulence Factor Subcategory | Organism |
| --- | --- | --- | --- | --- |
| Endotoxins | lpxC | lpxC | LPS | *Acinetobacter baumannii 1656-2* |
|  | rfbB | dTDP-glucose 4,6-dehydratase RmlB | LPS | *Legionella pneumophila subsp. pneumophila str. Philadelphia 1* |
|  | waaC | 3-deoxy-D-manno-octulosonic-acid (KDO) transferase | LPS | *Pseudomonas aeruginosa PAO1* |
|  | waaF | heptosyltransferase I | LPS | *Pseudomonas aeruginosa PAO1* |
|  | lpxA | lpxA | LPS | *Acinetobacter baumannii 1656-2* |
|  | acpXL | acyl carrier protein | LPS | *Brucella melitensis bv. 1 str. 16M* |
|  | fabZ | (3R)-hydroxymyristoyl ACP dehydratase | LPS | *Brucella suis 1330* |
|  | lpxK | tetraacyldisaccharide 4'-kinase (lipid-A 4'-kinase) | LOS | *Haemophilus somnus 129PT* |
|  | msbA | fused lipid transporter subunits of ABC superfamily: membrane component/ATP-binding component | LOS | *Haemophilus influenzae PittEE* |
|  | orfM | putative deoxyribonucleotide triphosphate pyrophosphatase | LOS | *Haemophilus influenzae 86-028NP* |
|  | rfaD | ADP-L-glycero-D-manno-heptose-6-epimerase | LOS | *Haemophilus somnus 2336* |
|  | rfaE | ADP-heptose synthase | LOS | *Haemophilus somnus 129PT* |
|  | gmhA | phosphoheptose isomerase | LOS | *Campylobacter fetus subsp. fetus 82-40* |
| Capsule and other surface component | glnA1 | glutamine synthetase, type I | Glutamine synthesis | *Mycobacterium gilvum PYR-GCK** |
|  | ABK1_0088 | Putative NeuA | Capsule | *Acinetobacter baumannii 1656-2* |
|  | ACICU_00076 | pyridoxal phosphate-dependent enzyme | Capsule | *Acinetobacter baumannii ACICU* |
|  | ACICU_00080 | sialic acid synthase | Capsule | *Acinetobacter baumannii ACICU* |
|  | algC | phosphomannomutase AlgC | Alginate biosynthesis | *Pseudomonas aeruginosa PA7* |
|  | algU | RNA polymerase, sigma-24 subunit, ECF subfamily | Alginate regulation | *Pseudomonas mendocina ymp* |
|  | algW | HtrA-like protease AlgW | Alginate regulation | *Pseudomonas putida KT2440* |
|  | BC5263 | UDP-glucose 4-epimerase | Polysaccharide capsule | *Bacillus cereus ATCC 14579* |
|  | BC5275 | UTP--glucose-1-phosphate uridylyltransferase | Polysaccharide capsule | *Bacillus cereus ATCC 14579* |
|  | BJAB07104_00096 | putative UDP-glucose 6-dehydrogenase | Capsule | *Acinetobacter baumannii BJAB07104* |
|  | BJAB0868_00089 | Protein-tyrosine-phosphatase | Capsule | *Acinetobacter baumannii BJAB0868* |
|  | gtaB | UTP--glucose-1-phosphate uridylyltransferase | Polysaccharide capsule | *Bacillus thuringiensis str. Al Hakam* |
|  | kpsF | arabinose-5-phosphate isomerase | Capsule biosynthesis and transport | *Campylobacter jejuni RM1221* |
|  | manC | putative GDP-mannose pyrophosphorylase | Capsule I | *Burkholderia cenocepacia J2315* |
|  | mrsA/glmM | phosphoglucosamine mutase | Exopolysaccharide | *Haemophilus somnus 129PT* |
|  | oppF | oligopeptide ABC transporter, permease component | Capsule | *Mycoplasma mycoides subsp. mycoides SC str. PG1* |
|  | pgi | glucose-6-phosphate isomerase | Exopolysaccharide | *Haemophilus somnus 129PT* |
|  | rmlA | putative glucose-1-phosphate thymidyltransferase | Capsule | *Streptococcus mutans UA159* |
|  | uppS | undecaprenyl diphosphate synthase | Capsule | *Enterococcus faecium Aus0004* |
|  | YE105_C1500 | Mannose-1-phosphate guanylytransferase, ManC protein | O-antigen | *Yersinia enterocolitica subsp. palearctica 105.5R(r)* |
|  | ompA | outer membrane protein OmpA | Capsule | *Piscirickettsia salmonis* |
| Secretion system | dotH/IcmK | IcmK protein | Dot/Icm type IVB secretion system | *Legionella pneumophila str. Philadelphia 1* |
|  | dotG/IcmE | IcmE protein | Dot/Icm type IVB secretion system | *Legionella pneumophila str. Philadelphia 1* |
|  | dotF/IcmG | IcmG protein | Dot/Icm type IVB secretion system | *Legionella pneumophila str. Philadelphia 1* |
|  | lpg0472 | dotE/IcmC protein | Dot/Icm type IVB secretion system | *Legionella pneumophila str. Philadelphia 1* |
|  | dotD | lipoprotein DotD | Dot/Icm type IVB secretion system | *Legionella pneumophila str. Philadelphia 1* |
|  | dotC | dotC protein | Dot/Icm type IVB secretion system | *Legionella pneumophila str. Philadelphia 1* |
|  | dotB | ATPase | Dot/Icm type IVB secretion system | *Legionella pneumophila str. Philadelphia 1* |
|  | IcmT | IcmT protein | Dot/Icm type IVB secretion system | *Legionella pneumophila str. Philadelphia 1* |
|  | dotL/IcmO | IcmO protein | Dot/Icm type IVB secretion system | *Legionella pneumophila str. Philadelphia 1* |
|  | dotM/IcmP | IcmP protein | Dot/Icm type IVB secretion system | *Legionella pneumophila str. Philadelphia 1* |
|  | dotN/IcmJ | IcmJ protein | Dot/Icm type IVB secretion system | *Legionella pneumophila str. Philadelphia 1* |
|  | dotO/IcmB | IcmB protein | Dot/Icm type IVB secretion system | *Legionella pneumophila str. Philadelphia 1* |
|  | dotA | defect in organelle trafficking protein DotA | Dot/Icm type IVB secretion system | *Legionella pneumophila str. Philadelphia 1* |
|  | dotI/IcmL | IcmL protein | Dot/Icm type IVB secretion system | *Legionella pneumophila str. Philadelphia 1* |
|  | IcmV | intracellular multiplication protein IcmV | Dot/Icm type IVB secretion system | *Legionella pneumophila str. Philadelphia 1* |
|  | CbuG_0446 | hypothetical protein | T4SS effectors | *Coxiella burnetii CbuG_Q212* |
|  | CbuG_1738 | trans-2-enoyl-CoA reductase | T4SS effectors | *Coxiella burnetii CbuG_Q212* |
|  | CbuK_1823 | GatB/Yqey domain protein | T4SS effectors | *Coxiella burnetii CbuK_Q154* |
|  | coxH3 | alpha/beta hydrolase | T4SS effectors | *Coxiella burnetii CbuG_Q212* |
|  | hsiB2 | hypothetical protein | HSI-2 | *Pseudomonas aeruginosa PAO1* |
|  | hsiC2 | hypothetical protein | HSI-2 | *Pseudomonas aeruginosa PAO1* |
| Adherence, colonization and invasion factor | flhB | flagellar biosynthetic protein FlhB | Flagella | *Pseudomonas mendocina ymp* |
|  | ASA_1361 | SOJ-like and chromosome partitioning protein | Polar flagella | *Aeromonas salmonicida subsp. salmonicida A449* |
|  | cheA | chemotaxis protein CheA | Flagella | *Vibrio fischeri ES114* |
|  | cheV | chemotaxis protein | Deoxyhexose linking sugar, 209 Da capping structure | *Pseudomonas aeruginosa PAO1* |
|  | cheV | chemotaxis CheV | Flagella | *Vibrio parahaemolyticus RIMD 2210633* |
|  | cheW | chemotaxis protein CheW | Flagella | *Vibrio parahaemolyticus RIMD 2210633* |
|  | cheY | chemotaxis protein CheY | Flagella | *Vibrio cholerae O1 biovar El Tor str. N16961* |
|  | crc | catabolite repression control protein | type IV pili | *Pseudomonas aeruginosa PAO1* |
|  | fleN | flagellar synthesis regulator FleN | polar flagella | *Legionella pneumophila subsp. pneumophila str. Philadelphia 1* |
|  | fleQ | sigma54 specific transcriptional regulator, Fis family | Flagella | *Pseudomonas putida F1* |
|  | flgB | flagellar basal-body rod protein FlgB | Flagella | *Pseudomonas mendocina ymp* |
|  | flgC | flagellar basal body rod protein FlgC | Polar flagella | *Aeromonas salmonicida subsp. salmonicida A449* |
|  | flgD | flagellar basal-body rod modification protein FlgD | Flagella | *Pseudomonas aeruginosa PA7* |
|  | flgE | flagellar hook protein | Flagella | *Pseudomonas syringae pv. syringae B728a* |
|  | flgG | flagellar basal-body rod protein FlgG | Flagella | *Pseudomonas fluorescens Pf-5* |
|  | flgH | flagellar L-ring protein precursor FlgH | Flagella | *Pseudomonas aeruginosa PA7* |
|  | flgI | flagellar P-ring protein | Flagella | *Pseudomonas putida GB-1* |
|  | flhA | flagellar biosynthesis protein | Flagella | *Vibrio parahaemolyticus RIMD 2210633* |
|  | fliA | flagellar biosynthesis sigma factor FliA | Deoxyhexose linking sugar, 209 Da capping structure | *Pseudomonas aeruginosa PAO1* |
|  | fliG | flagellar motor protein | Flagella | *Pseudomonas fluorescens Pf-5* |
|  | fliI | flagellar protein export ATPase FliI | Polar flagella | *Aeromonas veronii B565* |
|  | fliM | flagellar motor switch protein FliM | ND | *Aeromonas hydrophila subsp. hydrophila ATCC 7966* |
|  | fliN | flagellar motor switch protein | Flagella | *Pseudomonas syringae pv. syringae B728a* |
|  | fliP | flagellar biosynthesis | Flagella | *Pseudomonas fluorescens SBW25* |
|  | fliQ | flagellar biosynthetic protein FliQ | Flagella | *Pseudomonas mendocina ymp* |
|  | fliR | flagellar biosynthetic protein FliR | Flagella | *Pseudomonas putida W619* |
|  | flmH | 3-oxoacyl-ACP reductase | Polar flagella | *Aeromonas hydrophila ML09-119* |
|  | flrC | FlrC; two-component response regulator | Flagella | *Vibrio fischeri ES114* |
|  | motA | flagellar motor protein | Flagella | *Vibrio vulnificus CMCP6* |
|  | motB | flagellar motor protein | Flagella | *Vibrio fischeri ES114* |
|  | ompA | outer membrane protein OmpA | Flagella | *Piscirickettsia salmonis* |
|  | pseB | polysaccharide biosynthesis protein | O-linked flagellar glycosylation | *Campylobacter jejuni subsp. jejuni 81-176* |
|  | rpoS | RNA polymerase sigma factor RpoS | type IV pili | *Pseudomonas aeruginosa PAO1* |
|  | rpoS | RNA polymerase sigma factor RpoS | type IV pili | *Pseudomonas aeruginosa PAO1* |
|  | PilZ | pilus assembly protein PilZ | type IV pili | *Piscirickettsia salmonis* |
|  | PilA | type IV pilus biogenesis protein PilA | type IV pili | *Piscirickettsia salmonis* |
|  | PilB | type IV pilus assembly protein PilB | type IV pili | *Piscirickettsia salmonis* |
|  | PilC | type IV pilus biogenesis protein PilC | type IV pili | *Piscirickettsia salmonis* |
|  | PilD | type IV leader peptidase PilD | type IV pili | *Piscirickettsia salmonis* |
|  | PilQ | type IV pilus secretin PilQ | type IV pili | *Piscirickettsia salmonis* |
|  | PilT | ATPase | type IV pili | *Piscirickettsia salmonis* |
|  | PilW | type IV pilus biogenesis/stability protein PilW | type IV pili | *Piscirickettsia salmonis* |
|  | gacA | two component transcriptional regulator, LuxR family | GacS/GacA two-component system | *Pseudomonas putida W619* |
|  | armR | two-component response regulator | Cpi-1a + Cpi-1 | *Chromobacterium violaceum ATCC 12472* |
| Stress response | clpP | ATP-dependent Clp protease proteolytic subunit | ClpP | *Listeria monocytogenes EGD-e* |
|  | clpC | endopeptidase Clp ATP-binding chain C | ClpC | *Listeria monocytogenes EGD-e* |
|  | relA | GTP pyrophosphokinase ((p)ppGpp synthetase I) stringent stress response RelA | RelA | *Legionella pneumophila str. Corby* |
|  | PSLF89_RS14200 | molecular chaperone DnaK | HSP70 | *Piscirickettsia salmonis* |
|  | PSLF89_RS14195 | molecular chaperone DnaJ | HSP40 | *Piscirickettsia salmonis* |
|  | htpB | molecular chaperone GroEL/GroES | Hsp60 | *Legionella longbeachae NSW150* |
|  | sodB | superoxide dismutase | SodB | *Legionella pneumophila subsp. pneumophila str. Philadelphia 1* |
|  | sodCI | superoxide dismutase precursor (Cu-Zn) | SodCI | *Salmonella enterica subsp. enterica serovar Typhimurium str. LT2* |
|  | katA | catalase | Catalase | *Neisseria meningitidis alpha14* |
| Enzymes | eno | Enolase, putative | Streptococcal enolase | *Streptococcus sanguinis SK36* |
|  | ndk | nucleoside diphosphate kinase | Nucleoside diphosphate kinase | *Mycobacterium smegmatis str. MC2 155* |
|  | panD | aspartate 1-decarboxylase | Pantothenate synthesis | *Mycobacterium abscessus subsp. bolletii 50594* |
|  | plr/gapA | glyceraldehyde-3-phosphate dehydrogenase | Streptococcal plasmin receptor/GAPDH | *Streptococcus pyogenes M1 GAS* |
| iron uptake | pvsE | putative diaminopimelate decarboxylase protein | vibrioferrin | *Vibrio parahaemolyticus RIMD 2210633* |
|  | fhuC | ferrichrome transport ATP-binding protein | siderophore | *Piscirickettsia salmonis* |
|  | feoB | ferrous iron transporter B | FeoAB | *Legionella pneumophila subsp. pneumophila str. Philadelphia 1* |
|  | fur | Transcriptional repressor of iron-responsive genes (Fur family) (ferric uptake regulator) | Fur | *Salmonella enterica subsp. enterica serovar Typhimurium str. LT2* |
|  | hemA | glutamyl-tRNA reductase | Heme biosynthesis | *Piscirickettsia salmonis* |
|  | hemB | Porphobilinogen synthase | Heme biosynthesis | *Haemophilus somnus 2336* |
|  | hemC | porphobilinogen deaminase | Heme biosynthesis | *Haemophilus somnus 2336* |
|  | hemD | Uroporphyrinogen III synthase | Heme biosynthesis | *Piscirickettsia salmonis* |
|  | hemE | uroporphyrinogen decarboxylase | Heme biosynthesis | *Haemophilus somnus 2336* |
|  | hemF | Coproporphyrinogen III oxidase | Heme biosynthesis | *Piscirickettsia salmonis* |
|  | hemH | ferrochelatase | Heme biosynthesis | *Piscirickettsia salmonis* |
|  | hemL | glutamate-1-semialdehyde-2,1-aminomutase | Heme biosynthesis | *Haemophilus somnus 2336* |
|  | hemN | coproporphyrinogen III oxidase | Heme biosynthesis | *Haemophilus influenzae 86-028NP* |
|  | hemN | coproporphyrinogen III oxidase | Heme biosynthesis | *Piscirickettsia salmonis* |
|  | hemO | protoheme IX farnesyltransferase | Heme biosynthesis | *Piscirickettsia salmonis* |
|  | hemY | protoporphyrinogen IX and coproporphyrinogen III oxidase | Heme biosynthesis | *Piscirickettsia salmonis* |
|  | SufA | iron-sulfur cluster assembly accessory protein SufA | Iron–sulfur cluster biosynthesis | *Piscirickettsia salmonis* |
|  | SufS | cysteine desulfurase | Iron–sulfur cluster biosynthesis | *Piscirickettsia salmonis* |
|  | dps | ferritin-like protein | iron storage | *Piscirickettsia salmonis* |
|  | ExbB | biopolymer transport protein ExbB | Iron transport | *Piscirickettsia salmonis* |
|  | pvsB | Vibrioferrin amide bond forming protein | Vibrioferrin | *Piscirickettsia salmonis* |
|  | pvsD | Vibrioferrin amide bond forming protein | Vibrioferrin | *Piscirickettsia salmonis* |
|  | TonB | TonB-dependent siderophore receptor | Iron transport | *Piscirickettsia salmonis* |
