# Supplementary material for: Comparative Pan-Genome Analysis of Piscirickettsia salmonis Reveals Genomic Divergences within Genogroups
Source: Front Cell Infect Microbiol. 2017 Oct 31;7:459. doi: 10.3389/fcimb.2017.00459 (PMC5671498; doi:10.3389/fcimb.2017.00459)
Supplement: Supplementary Table 2 — Information on ribosomal operons in the 19 complete genomes of P. salmonis. [file Table2.DOCX]

**Supplementary Table 2**

| Strain | Start | End | Length (nt) | Direction |
| --- | --- | --- | --- | --- |
| AY3800B | 88878 | 93939 | 5061 | + |
|  | 236806 | 241868 | 5062 | + |
|  | 1740199 | 1745260 | 5061 | - |
|  | 2324253 | 2329589 | 5336 | - |
|  | 2507314 | 2512375 | 5061 | - |
|  | 3000183 | 3005520 | 5337 | - |
| AY3864B | 88878 | 93939 | 5061 | + |
|  | 236806 | 241868 | 5062 | + |
|  | 1740200 | 1745261 | 5061 | - |
|  | 2324254 | 2329590 | 5336 | - |
|  | 2507315 | 2512376 | 5061 | - |
|  | 3000184 | 3005521 | 5337 | - |
| AY6297B | 88878 | 93939 | 5061 | + |
|  | 236806 | 241868 | 5062 | + |
|  | 1740196 | 1745257 | 5061 | - |
|  | 2324250 | 2329586 | 5336 | - |
|  | 2507309 | 2512370 | 5061 | - |
|  | 3000179 | 3005516 | 5337 | - |
| AY6492A | 175279 | 180614 | 5335 | + |
|  | 1514720 | 1520055 | 5335 | + |
|  | 2216880 | 2221941 | 5061 | - |
|  | 2270961 | 2276025 | 5064 | - |
|  | 2791560 | 2796621 | 5061 | - |
|  | 2945448 | 2950508 | 5060 | - |
| AY6532B | 88875 | 93936 | 5061 | + |
|  | 236802 | 241864 | 5062 | + |
|  | 1739075 | 1744136 | 5061 | - |
|  | 2323177 | 2328513 | 5336 | - |
|  | 2506232 | 2511293 | 5061 | - |
|  | 2999090 | 3004425 | 5335 | - |
| PSCGR02 | 88879 | 93940 | 5061 | + |
|  | 236807 | 241869 | 5062 | + |
|  | 1755271 | 1760332 | 5061 | - |
|  | 2340505 | 2345841 | 5336 | - |
|  | 2523546 | 2528607 | 5061 | - |
|  | 3016735 | 3022072 | 5337 | - |
| LF-89 | 89167 | 94228 | 5061 | + |
|  | 237096 | 242157 | 5061 | + |
|  | 1738272 | 1743333 | 5061 | - |
|  | 2321896 | 2327232 | 5336 | - |
|  | 2504695 | 2509757 | 5062 | - |
|  | 2996321 | 3001659 | 5338 | - |
| PM15972A1 | 175279 | 180614 | 5335 | + |
|  | 1514741 | 1520076 | 5335 | + |
|  | 2236504 | 2241565 | 5061 | - |
|  | 2290585 | 2295649 | 5064 | - |
|  | 2811280 | 2816341 | 5061 | - |
|  | 2965171 | 2970231 | 5060 | - |
| PM21567A | 175272 | 180607 | 5335 | + |
|  | 1514706 | 1520041 | 5335 | + |
|  | 2216862 | 2221923 | 5061 | - |
|  | 2270941 | 2276005 | 5064 | - |
|  | 2791646 | 2796707 | 5061 | - |
|  | 2945536 | 2950596 | 5060 | - |
| PM22180B | 89166 | 94227 | 5061 | + |
|  | 237095 | 242157 | 5062 | + |
|  | 1739780 | 1744841 | 5061 | - |
|  | 2323175 | 2328511 | 5336 | - |
|  | 2505963 | 2511024 | 5061 | - |
|  | 2997607 | 3002944 | 5337 | - |
| PM23019A | 173230 | 178565 | 5335 | + |
|  | 748964 | 754028 | 5064 | + |
|  | 806065 | 811129 | 5064 | + |
|  | 1524007 | 1529342 | 5335 | - |
|  | 2804518 | 2809583 | 5065 | - |
|  | 2958407 | 2963471 | 5064 | - |
| PM25344B | 89186 | 94247 | 5061 | + |
|  | 237151 | 242212 | 5061 | + |
|  | 1740587 | 1745646 | 5059 | - |
|  | 2324193 | 2329528 | 5335 | - |
|  | 2507016 | 2512075 | 5059 | - |
|  | 2998838 | 3004175 | 5337 | - |
| PM31429B | 88878 | 93939 | 5061 | + |
|  | 236806 | 241868 | 5062 | + |
|  | 1739907 | 1744968 | 5061 | - |
|  | 2323168 | 2328504 | 5336 | - |
|  | 2505955 | 2511016 | 5061 | - |
|  | 3005126 | 3010463 | 5337 | - |
| PM32597B1 | 89166 | 94227 | 5061 | + |
|  | 237094 | 242156 | 5062 | + |
|  | 1739776 | 1744837 | 5061 | - |
|  | 2323171 | 2328507 | 5336 | - |
|  | 2505959 | 2511020 | 5061 | - |
|  | 2997605 | 3002942 | 5337 | - |
| PM37984A | 173241 | 178576 | 5335 | + |
|  | 749007 | 754071 | 5064 | + |
|  | 806111 | 811175 | 5064 | + |
|  | 1524111 | 1529446 | 5335 | - |
|  | 2796294 | 2801359 | 5065 | - |
|  | 2950189 | 2955253 | 5064 | - |
| PM49811B | 88877 | 93938 | 5061 | + |
|  | 236803 | 241865 | 5062 | + |
|  | 1739831 | 1744892 | 5061 | - |
|  | 2323078 | 2328414 | 5336 | - |
|  | 2505860 | 2510921 | 5061 | - |
|  | 3005019 | 3010356 | 5337 | - |
| PM51819A | 171819 | 177154 | 5335 | + |
|  | 778585 | 783649 | 5064 | + |
|  | 834994 | 840055 | 5061 | + |
|  | 1613303 | 1618640 | 5337 | + |
|  | 2888926 | 2893987 | 5061 | - |
|  | 3045015 | 3050075 | 5060 | - |
| PM58386B | 88878 | 93939 | 5061 | + |
|  | 237093 | 242155 | 5062 | + |
|  | 1740419 | 1745480 | 5061 | - |
|  | 2323691 | 2329027 | 5336 | - |
|  | 2506477 | 2511538 | 5061 | - |
|  | 3005645 | 3010982 | 5337 | - |
| PSCGR01 | 89165 | 94226 | 5061 | + |
|  | 237093 | 242155 | 5062 | + |
|  | 1712142 | 1717203 | 5061 | - |
|  | 2295863 | 2301199 | 5336 | - |
|  | 2478660 | 2483721 | 5061 | - |
|  | 2970323 | 2975660 | 5337 | - |
