# Supplementary material for: Comparative Pan-Genome Analysis of Piscirickettsia salmonis Reveals Genomic Divergences within Genogroups
Source: Front Cell Infect Microbiol. 2017 Oct 31;7:459. doi: 10.3389/fcimb.2017.00459 (PMC5671498; doi:10.3389/fcimb.2017.00459)
Supplement: Supplementary Table 3 — Annotation of the genetic neighborhood of each ribosomal operon. Each ID corresponds a gene represented in the Supplementary Figure 5. [file Table3.DOCX]

**Supplementary Table 3**

| ID | Accession number | Annotation |
| --- | --- | --- |
| 1 | WP_027243063.1 | Hydroxyacylglutathione hydrolase |
| 2 | ALB21284.1 | Outer membrane protein/outer membrane enzyme PagP |
| 3 | WP_054300546.1 (partial) | Transposase |
| 4 | ALA26437.1 (partial) | NUDIX hydrolase |
| 5 | WP_017376237.1 | SDR family oxidoreductase |
| 6 | WP_027242703.1 | phosphatase PAP2 family protein |
| 7 | KLV35471.1 | protein tyrosine phosphatase |
| 8 | KLV35466.1 | polysaccharide biosynthesis/export family protein |
| 9 | ALB21291.1 | Exopolysaccharide synthesis protein |
| 10 | WP_036771610.1 | UTP--glucose-1-phosphate uridylyltransferase |
| 11 | KLV36187.1 | nitronate monooxygenase |
| 12 | WP_017377637.1 | hypothetical protein |
| 13 | ALB21430.1 | transporter associated domain protein |
| 14 | WP_017377639.1 | signal recognition particle protein |
| 15 | WP_036772069.1 | paraquat-inducible A family protein |
| 16 | KLV35713.1 | Na+:H+ dicarboxylate symporter |
| 17 | WP_017376226.1 | bifunctional biotin |
| 18 | WP_048875884.1 | type III pantothenate kinase |
| 19 | ALB21221.1 | transposase |
| 20 | ALB22894.1 (partial) | transposase |
| 21 | WP_027243180.1 | phosphoesterase |
| 22 | KLV35278.1 | sodium:proton antiporter |
| 23 | WP_026063485.1 | thioesterase |
| 24 | WP_036772592.1 | hypothetical protein |
| 25 | WP_026063486.1 | cupin |
| 26 | KLV34743.1 | aminotransferase |
| 27 | WP_027243221.1 | cytochrome d terminal oxidase subunit I |
| 28 | WP_027243222.1 | cytochrome d ubiquinol oxidase subunit II |
| 29 | ALB22882.1 | transposase |
| 30 | ALB22894.1 | transposase |
| 31 | KLV35721.1 | BadM/Rrf2 family transcriptional regulator |
| 32 | WP_017377990.1 | SIS domain-containing protein |
| 33 | KLV35722.1 | MFS sugar transporter |
| 34 | KLV35723.1 | ATPase |
| 35 | KLV35724.1 | GlcNAc-PI de-N-acetylase |
| 36 | KLV34328.1 | peptidylprolyl isomerase |
| 37 | ALB23465.1 | DSBA oxidoreductase |
| 38 | ALB23464.1 | NADH dehydrogenase |
| 39 | KLV34331.1 | SAM-dependent methyltransferase |
| 40 | ALB23462.1 | mercuric resistance operon regulatory protein |
| 41 | KLV35614.1 | ABC transporter permease |
| 42 | KLV35615.1 | peptide ABC transporter ATP-binding protein |
| 43 | KLV35616.1 | peptide ABC transporter ATP-binding protein |
| 44 | KLV35618.1 | na+ dependent nucleoside transporter family protein |
| 45 | ALB23659.1 | cro/C1-type HTH DNA-binding domain protein |
| 46 | ALB23658.1 | methyl-accepting chemotaxis sensory transducer |
| 47 | ALB23657.1 | transposase |
| 48 | ALB22183.1 | integrase |
| 49 | WP_027242570.1 | tRNA nucleotidyltransferase |
| 50 | WP_017376020.1 | tRNA (N6-isopentenyl adenosine(37)-C2)-methylthiotransferase MiaB |
| 51 | ALB24174.1 | ppx/GppA phosphatase family protein |
| 52 | WP_016210045.1 | thiol reductase thioredoxin |
| 53 | WP_026063646.1 | transcription termination factor Rho |
| 54 | WP_048876055.1 | transposase |
| 55 | ALB24170.1 | S-adenosyl-L-homocysteine hydrolase NAD- binding protein |
| 56 | KLV36492.1 | membrane protein |
| 57 | ALB24168.1 | LysR substrate binding domain protein |
| 58 | WP_027242687.1 | hypothetical protein |
| 59 | KLV36489.1 | diacylglycerol kinase |
| 60 | KLV36488.1 | taurine ABC transporter ATP-binding protein |
| 61 | ALA26440.1 | transposase |
| 62 | ALA26439.1 | transposase |
| 63 | WP_059372539.1 (partial) | transposase |
| 64 | WP_016212445.1 | transposase |
| 65 | WP_054300340.1 | transposase |
| 66 | ALA26535.1 | integrase (plasmid) |
| 67 | ALB22778.1 | transposase |
| 68 | WP_036777175.1 | peptide ABC transporter ATP-binding protein |
| 69 | WP_032126219.1 | peptide ABC transporter ATP-binding protein |
| 70 | KLV35617.1 | RNA-binding protein |
| 71 | WP_016209548.1 | na+ dependent nucleoside transporter family protein |
| 72 | WP_016209571.1 | cro/C1-type HTH DNA-binding domain protein |
| 73 | WP_016211370.1 | rrf2 family protein |
| 74 | WP_016211369.1 | SIS domain protein |
| 75 | WP_016211368.1 | MFS transporter sugar porter family protein |
| 76 | WP_016211371.1 | badF/BadG/BcrA/BcrD ATPase |
| 77 | WP_016211372.1 | GlcNAc-PI de-N-acetylase |
| 78 | ALA24254.1 | transposase |
| 79 | ALA25758.1 | AAA domain protein |
| 80 | WP_016211411.1 | rRNA maturation RNase YbeY |
| 81 | ERL62815.1 | polyphosphate kinase 1 |
