# Supplementary material for: Comparative Pan-Genome Analysis of Piscirickettsia salmonis Reveals Genomic Divergences within Genogroups
Source: Front Cell Infect Microbiol. 2017 Oct 31;7:459. doi: 10.3389/fcimb.2017.00459 (PMC5671498; doi:10.3389/fcimb.2017.00459)
Supplement: Supplementary file 4 [file Image1.PDF]

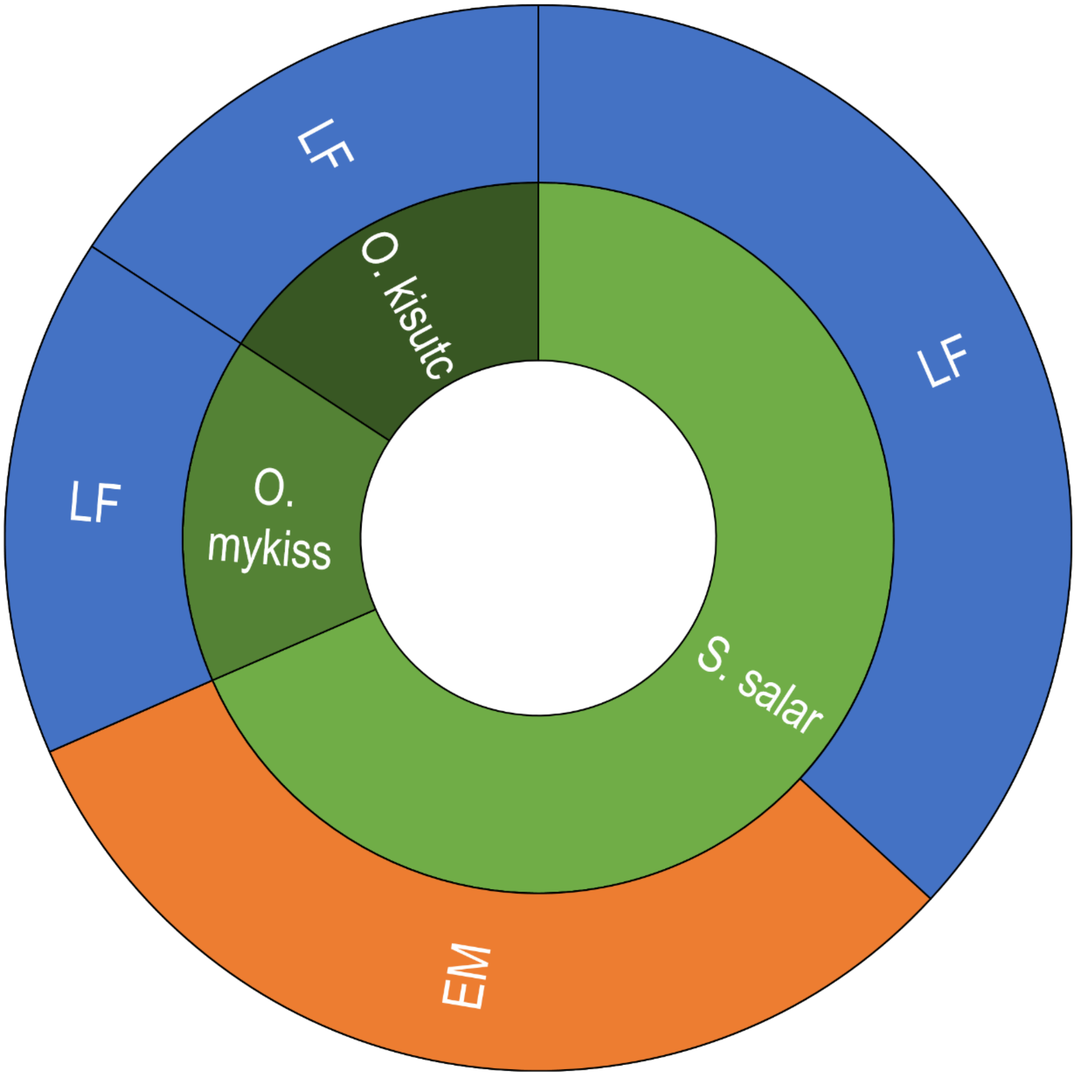

Supplementary Figure 1. Genogroups distribution in salmonids hosts. The nineteen fully sequenced strains from Chilean salmon industry where isolated from 1989 to 2015, the percentage of each genogroups in the different hosts where *S. salar* 68.4% (6 EM strains and 7 LF strains); *O. kisutch* 15.8% (3 LF strains); *O. mykiss* 15.8% (3 LF strains).
