# Supplementary material for: Comparative Pan-Genome Analysis of Piscirickettsia salmonis Reveals Genomic Divergences within Genogroups
Source: Front Cell Infect Microbiol. 2017 Oct 31;7:459. doi: 10.3389/fcimb.2017.00459 (PMC5671498; doi:10.3389/fcimb.2017.00459)
Supplement: Supplementary file 5 [file Image2.PDF]

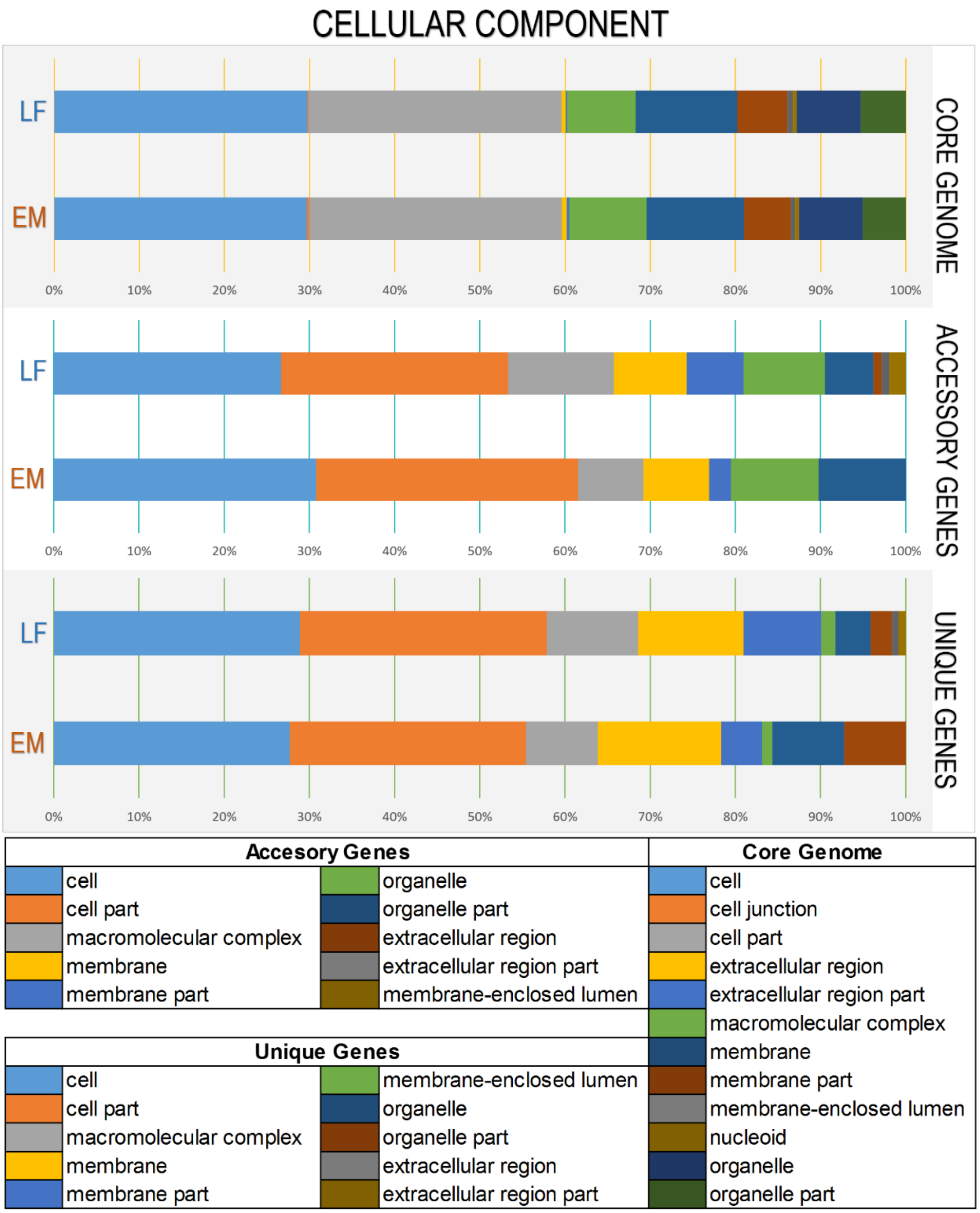

Supplementary Figure 2: Variation of functional annotation of pangenome component by Gene Ontology. The figure shows the variation between two genogroups through the level two of "Cellular Component", using the annotation file (see material and methods) and combined graph of Blast2GO. The input data set is separated into 3 parts, the core-genome, accessory genome and unique genes. Each bar represents a genogroup and colors inside the bar represents a function of the genes contained in each section.
