# Supplementary material for: Comparative Pan-Genome Analysis of Piscirickettsia salmonis Reveals Genomic Divergences within Genogroups
Source: Front Cell Infect Microbiol. 2017 Oct 31;7:459. doi: 10.3389/fcimb.2017.00459 (PMC5671498; doi:10.3389/fcimb.2017.00459)
Supplement: Supplementary file 7 [file Image4.PDF]

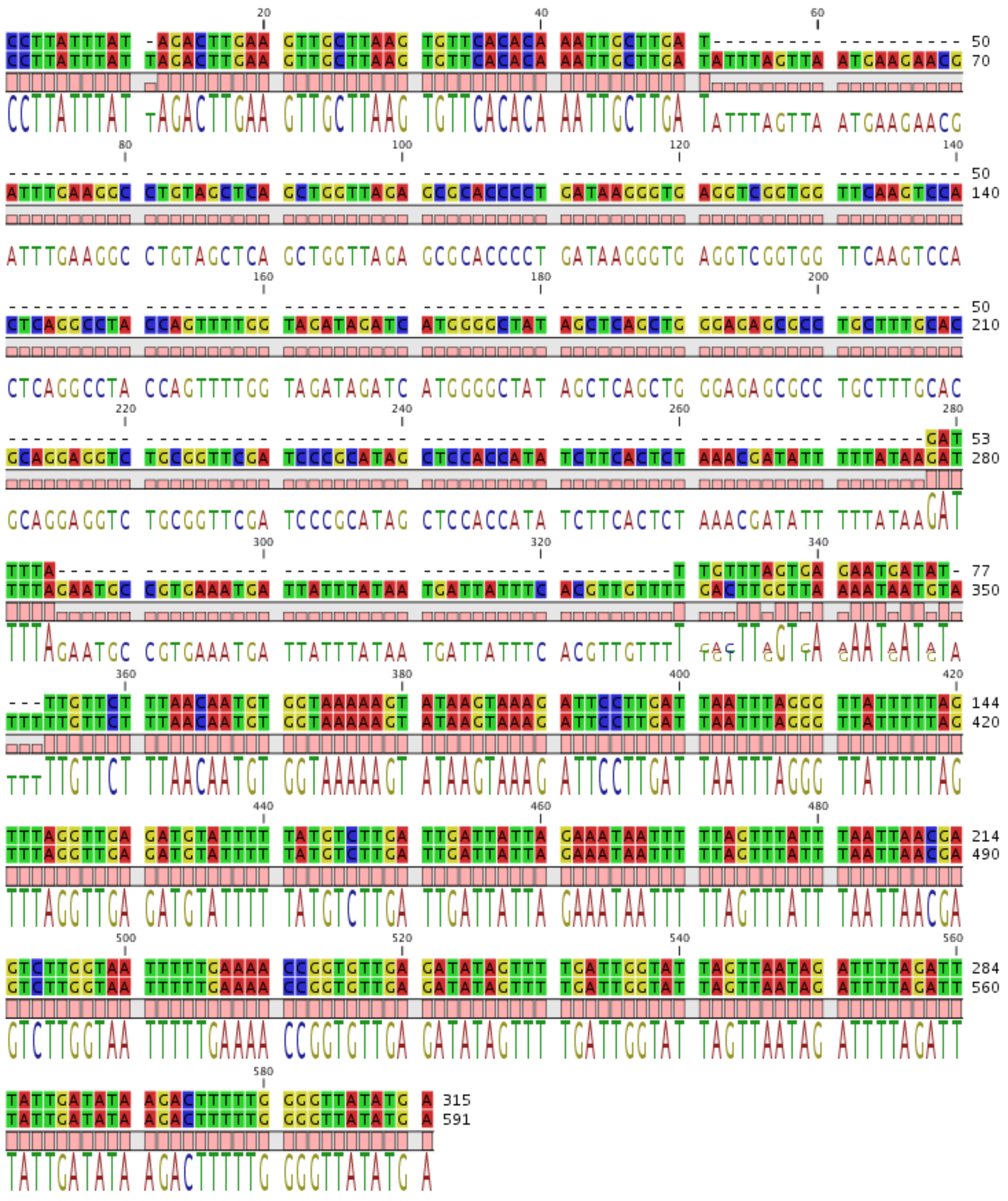

Supplementary Figure 4: Alignment of the ITS region between operons A and B from *Piscirickettsia salmonis LF-89*. Red dotted lines represent the region where the new fragment was inserted inside ITS region. The representation was obtained through CLC Genomics Workbench 8.0.3 (https://www.qiagenbioinformatics.com/).
