# Supplementary material for: Comparative Pan-Genome Analysis of Piscirickettsia salmonis Reveals Genomic Divergences within Genogroups
Source: Front Cell Infect Microbiol. 2017 Oct 31;7:459. doi: 10.3389/fcimb.2017.00459 (PMC5671498; doi:10.3389/fcimb.2017.00459)
Supplement: Supplementary file 8 [file Image5.PDF]

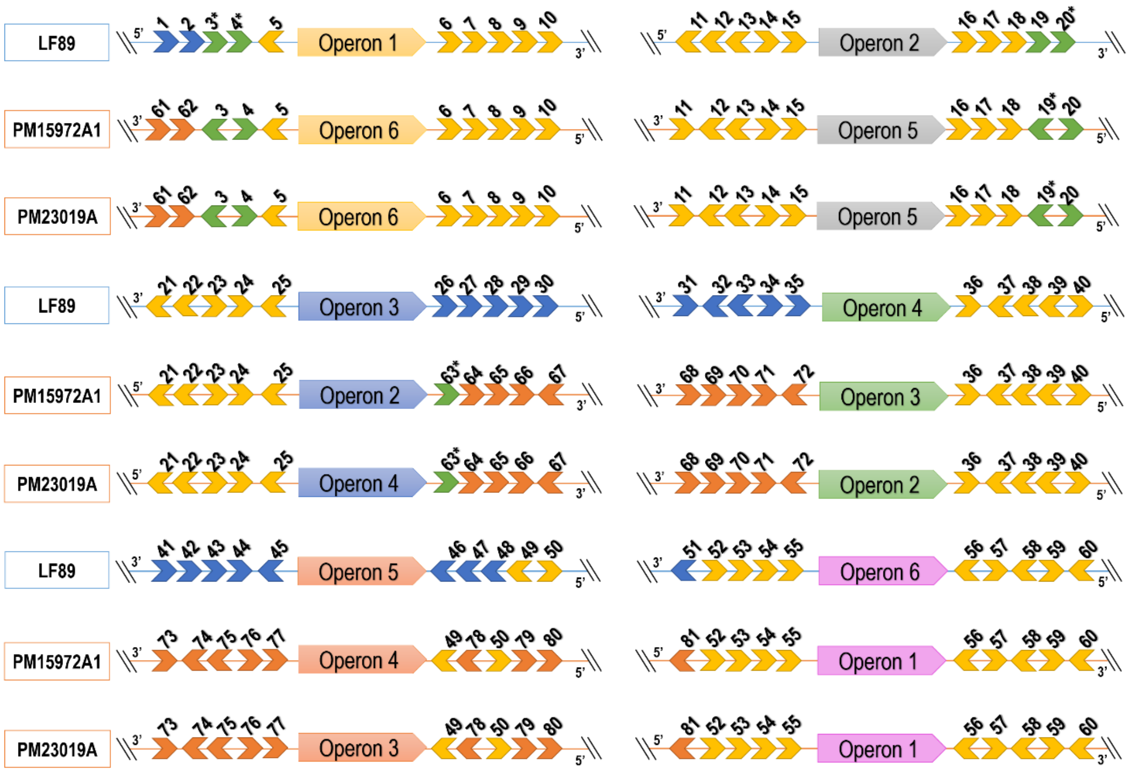

Supplementary Figure 5: Rearrangements details between the reference strain LF-89 and the most divergent complete genome from strain PM15972A1 and PM23019A (EM90-like). Comparison between neighbored genes located on the vicinity of each operon. Each gene was classified by different colors according to different characteristics. Yellow blocks represent genes conserved within strains. Blue blocks represent LF-89 genes, absent in PM15972A1. Orange blocks represents PM15972A1 genes, absent in LF-89. Green blocks represent interrupted genes (* indicates the truncated genes in each strain). The representation was ordered with respect to the directionality of each region. Annotation information in Supplementary table 3.
