# Supplementary figures and images for: Comparative Pan-Genome Analysis of Piscirickettsia salmonis Reveals Genomic Divergences within Genogroups
Source: Front Cell Infect Microbiol. 2017 Oct 31;7:459. doi: 10.3389/fcimb.2017.00459 (PMC5671498; doi:10.3389/fcimb.2017.00459)

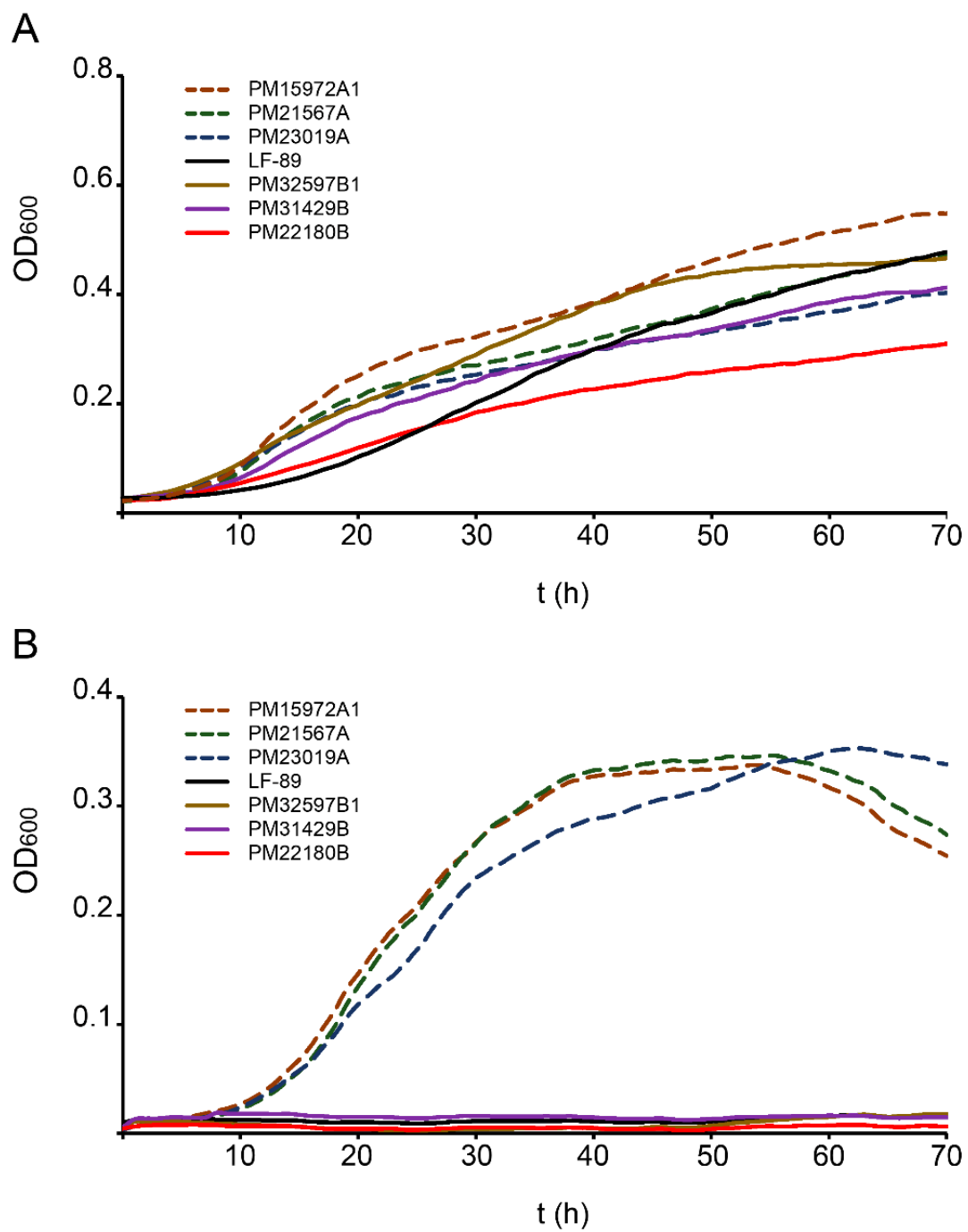

**Supplementary Figure 6:** Growth kinetics in ADL-PSB at 18 °C (A) and 22 °C (B) of some strains in ours work.

Supplement: Supplementary file 9 [file Image6.PDF]

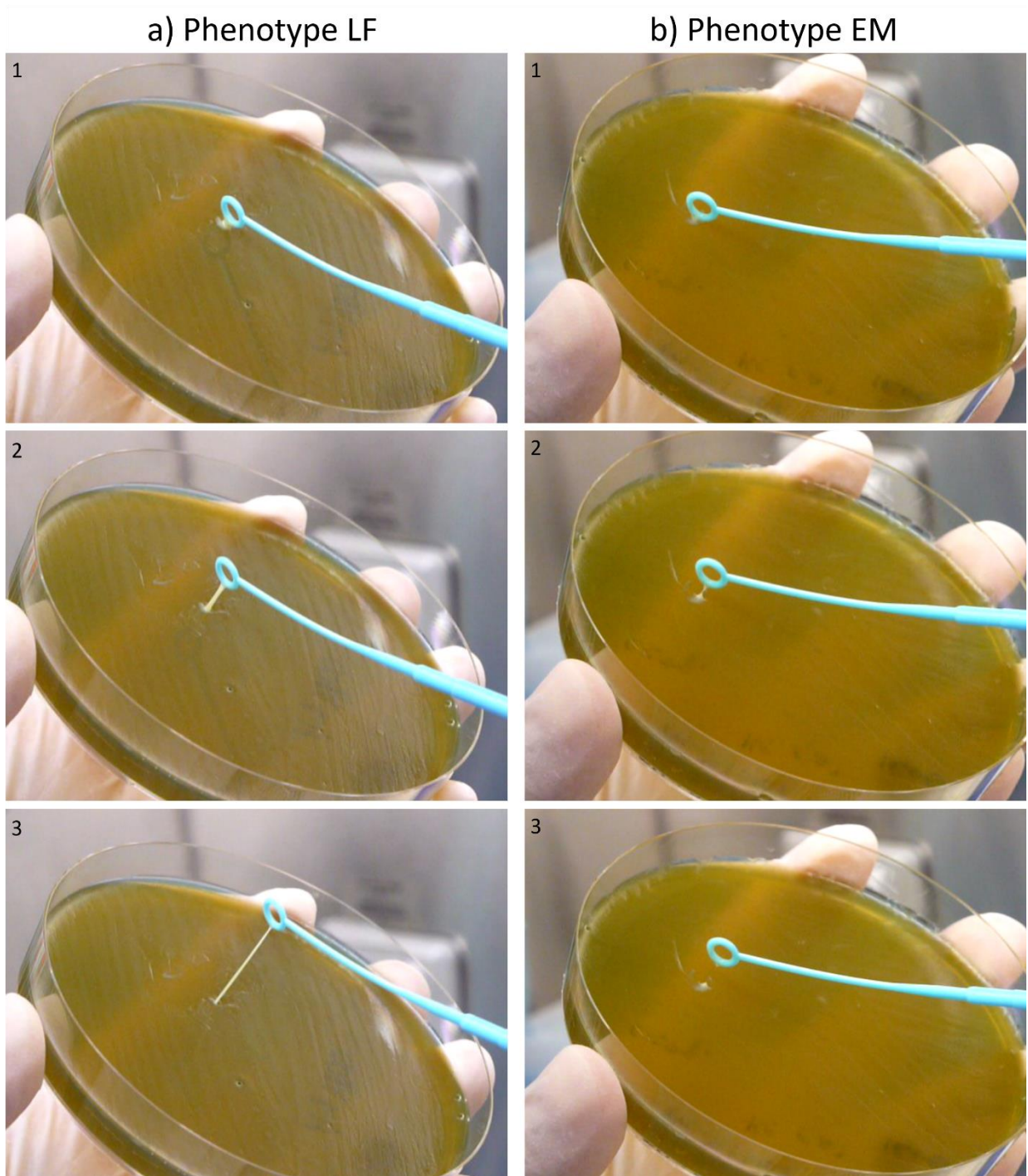

**Supplementary Figure 7:** Colony phenotypes of *P. salmonis* genogroups.

Supplement: Supplementary file 10 [file Image7.PDF]
